# Worldwide variations in artificial skyglow

**DOI:** 10.1038/srep08409

**Published:** 2015-02-12

**Authors:** Christopher C. M. Kyba, Kai Pong Tong, Jonathan Bennie, Ignacio Birriel, Jennifer J. Birriel, Andrew Cool, Arne Danielsen, Thomas W. Davies, Peter N. den Outer, William Edwards, Rainer Ehlert, Fabio Falchi, Jürgen Fischer, Andrea Giacomelli, Francesco Giubbilini, Marty Haaima, Claudia Hesse, Georg Heygster, Franz Hölker, Richard Inger, Linsey J. Jensen, Helga U. Kuechly, John Kuehn, Phil Langill, Dorien E. Lolkema, Matthew Nagy, Miguel Nievas, Nobuaki Ochi, Emil Popow, Thomas Posch, Johannes Puschnig, Thomas Ruhtz, Wim Schmidt, Robert Schwarz, Axel Schwope, Henk Spoelstra, Anthony Tekatch, Mark Trueblood, Constance E. Walker, Michael Weber, Douglas L. Welch, Jaime Zamorano, Kevin J. Gaston

**Affiliations:** 1 Leibniz-Institute for Freshwater Ecology and Inland Fisheries; 2 Institute for Space Sciences, Freie Universität Berlin, Berlin, Germany; 3 Deutsches GeoForschungsZentrum Potsdam, Potsdam, Germany; 4 Institut für Umweltphysik, Universität Bremen, Bremen, Germany; 5 Environment and Sustainability Institute, University of Exeter, Penryn, UK; 6 Morehead State University, Morehead, USA; 7 The Heights Observatory, Adelaide; 8 Unaffiliated Citizen Scientist; 9 National Institute for Public Health and the Environment, Bilthoven, The Netherlands; 10 Light Pollution Science and Technology Institute (ISTIL), Thiene, Italy; 11 Attivarti.org, Torniella, Italy; 12 Landesamt für Umwelt, Gesundheit und Verbraucherschutz Brandenburg, Germany; 13 National Optical Astronomy Observatory, Tucson, USA; 14 Utah State University, Logan, USA; 15 Rothney Astrophysical Observatory, University of Calgary, Calgary, Canada; 16 Department of Physics, University of Alberta, Edmonton, Canada; 17 Departamento de Astrofísica y Ciencias de la Atmósfera, Universidad Complutense de Madrid, Madrid, Spain; 18 Faculty of Business Administration, Toyo University, Tokyo, Japan; 19 Leibniz-Institut für Astrophysik Potsdam (AIP), Potsdam, Germany; 20 Institut für Astrophysik, Vienna, Austria; 21 Sotto le Stelle, Utrecht, The Netherlands; 22 Lumineux Consult, Arnhem, The Netherlands; 23 Unihedron, Grimsby, Canada; 24 Winer Observatory, Sonoita, USA; 25 Department of Physics and Astronomy, McMaster University, Hamilton, Canada

## Abstract

Despite constituting a widespread and significant environmental change, understanding of artificial nighttime skyglow is extremely limited. Until now, published monitoring studies have been local or regional in scope, and typically of short duration. In this first major international compilation of monitoring data we answer several key questions about skyglow properties. Skyglow is observed to vary over four orders of magnitude, a range hundreds of times larger than was the case before artificial light. Nearly all of the study sites were polluted by artificial light. A non-linear relationship is observed between the sky brightness on clear and overcast nights, with a change in behavior near the rural to urban landuse transition. Overcast skies ranged from a third darker to almost 18 times brighter than clear. Clear sky radiances estimated by the World Atlas of Artificial Night Sky Brightness were found to be overestimated by ~25%; our dataset will play an important role in the calibration and ground truthing of future skyglow models. Most of the brightly lit sites darkened as the night progressed, typically by ~5% per hour. The great variation in skyglow radiance observed from site-to-site and with changing meteorological conditions underlines the need for a long-term international monitoring program.

The introduction of artificial light has caused an unprecedented disruption to the nighttime environment over large areas of the Earth. Daily, seasonal, and lunar cycles of light that had previously been rather invariant for millennia have been dramatically altered by the spread of both public and private nighttime lighting, much of it associated with the global network of ~18 million kilometers of paved roads^1^. These changes have brought many human benefits, most importantly extending the hours available for productive work and social activity, but this gain has come at some cost. Artificial light at night has significant negative impacts for wildlife and ecosystems^2^,^3^,^4^,^5^,^6^, and evidence of deleterious consequences for human health and wellbeing continues to accumulate^7^,^8^,^9^,^10^.

Much recent attention has been paid to the impacts of exposure to direct emissions of artificial light at night^11^,^12^,^13^,^14^,^15^,^16^. In contrast, much less is known about the environmental consequences of indirect light exposure^17^. Light which is reflected or directly emitted upwards can be scattered back to Earth by atmospheric constituents, causing skyglow. This raises the overall background nighttime light level over vast areas, and can screen out celestial signals from individual stars^18^,^19^, the Milky Way^20^, and the polarization pattern of the moon^21^. The known and hypothesized effects of skyglow are diverse, and include changes to the time partitioning patterns of animals^4^,^22^; loss of key nighttime navigation signals for species^23^,^24^; changes in predator-prey relationships^25^,^26^; loss of human cultural experiences associated with naturally lit night skies and thought by some to be profound^27^; and difficulties with the siting and operation of astronomical telescopes^28^.

Until recently, understanding of typical levels and patterns of skyglow has been extremely poor. The invention of robust and easy to use light meters like the “Sky Quality Meter” (SQM; Unihedron), however, has resulted in a boom in skyglow measurement^29^,^30^,^31^,^32^,^33^,^34^,^35^,^36^,^37^,^38^,^39^,^40^. This has led to some understanding of patterns on a local scale, and emphasized the need for comparative studies across different regions. For example, sky radiance can have contrasting trends during a single night in nearby locations. The sky over a city often becomes progressively darker over the course of the night (Fig. 1A). In contrast, at nearby rural locations, the sky becomes brighter as the moon rises. Clouds play a key role in determining sky radiance in locations that are primarily artificially lit (Fig. 1B). Because water droplets are almost non-absorbing at visible wavelengths, clouds can return a large fraction of city light to the ground. Skyglow can vary extremely rapidly in urban locations as clouds pass over and then away from the site. On partly cloudy nights, skyglow is sometimes observed to switch rapidly between two levels as small clouds pass over the site^29^,^30^,^37^.

In this study, for the first time we bring together data obtained from SQMs at 44 sites around the globe to address four key questions about skyglow: 1) what levels of sky radiance are currently experienced worldwide, and how do these compare to the levels experienced under celestial light only? 2) do model-based predictions of skyglow on clear nights match the observational data? 3) is there a simple relation between the amplification of skyglow by clouds and the level of skyglow on clear days? 4) how do levels of skyglow change throughout the night?

## Results

### Observed sky radiance

Night sky radiances were measured using SQMs during astronomical night (see methods). Radiance ranged over almost four orders of magnitude, from darkest values of 23.24 mag*_SQM_*/arcsec^2^ at Kitt Peak, USA (1^st^ percentile), to brightest values of 13.26 mag*_SQM_*/arcsec^2^ at Schipluiden, Netherlands (99^th^ percentile). In “natural sky units” (radiance relative to an assumed natural radiance of 21.6 mag*_SQM_*/arcsec^2^, see methods), the range was 0.22–2200 NSU. Before the introduction of anthropogenic light, the radiance of a relatively large patch of sky near zenith on moon-free nights is likely to have been nearly always within the range 20 (galactic center near zenith) to 24 mag/arcsec^2^ (very thick clouds), or 0.1–4.3 NSU. The clear sky radiance at most sites was considerably larger than the typical signal expected from celestial sources alone (21.4–21.6 mag*_SQM_*/arcsec^2^, 1–1.2 NSU), so from the viewpoint of stellar visibility, nearly all of the sites can be said to suffer from light pollution.

Artificial skyglow is approximately equal in radiance to natural sources at 20.85 mag*_SQM_*/arcsec^2^ (NSU = 2). Anthropogenic skyglow dominated over celestial light on clear nights at midnight at 18 of the 22 sites for which cloud cover data were available (Table S3). The sky was brighter than 2 NSU more than 95% of the time at 30 sites. Remarkably, at 7 of these sites, the sky was at least 10 times brighter than natural 95% of the time.

Overcast moonlit nights provide an opportunity to compare artificial skyglow levels to an equivalent natural source of diffuse light. Moonlight increased the median radiance of the overcast (8 okta) night sky at each location examined (Table S4). This effect was very large at the sites with natural night skies (more than a factor of 10 at Schiermonnikoog, The Netherlands), but small (5–20%) at the brightest sites. At sites with little artificial skyglow, the rising moon rapidly brightened the overcast night sky, to a maximum of ~16 mag*_SQM_*/arcsec^2^ at the unpolluted Schiermonnikoog site (Fig. S7). For comparison, Garstang^41^ predicted that on a cloudy night a full moon at 64° elevation would produce a maximum overcast sky radiance of about 15.5 mag*_SQM_*/arcsec^2^. In contrast, at urban sites the lunar cycle was no longer visible on overcast nights, and the sky brightness instead mainly depended on cloud properties (Fig. S7).

### Comparison of clear sky data to skyglow simulations

**Figure 2:**
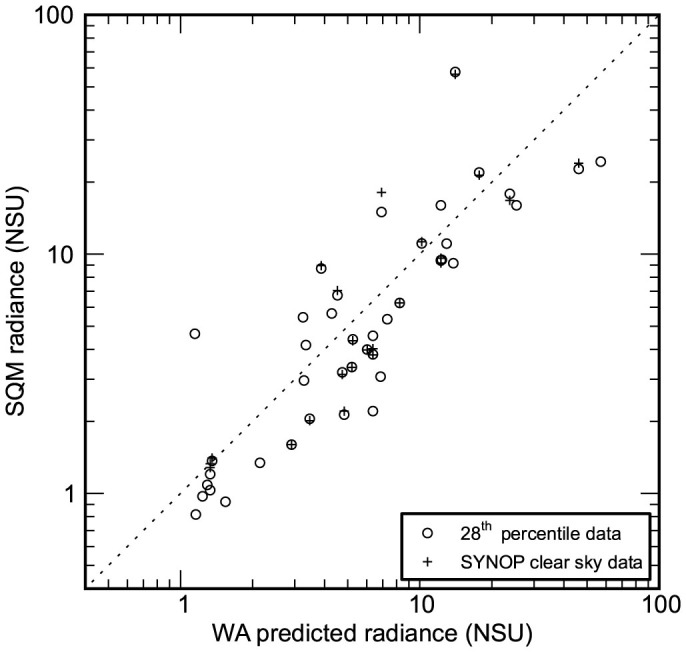
Comparison of clear sky observations to World Atlas values. Radiances are plotted in “natural sky units”. Circles indicate the 28^th^ percentile brightness at each site, and crosses show the median radiance for sites with SYNOP data. Observations that perfectly matched the prediction would lie on the dashed line.

The World Atlas of Artificial Night Sky Brightness remains the only skyglow model with global coverage^20^. It was found to overestimate the sky brightness observed at the study sites by a factor of about 25% in NSU (Fig. 2, Table S3). After correcting for this, the standard deviation of the difference between the observed and estimated values was ~40% in NSU. These results are similar to those found in a recent study which compared handheld SQM observations by citizen scientists to the World Atlas^36^. Two likely sources of the relatively small difference between the data and model are differences in atmospheric transparency and bias due to snow cover in the satellite data used to produce the Atlas.

### Relationship between overcast and clear sky radiance

**Figure 3:**
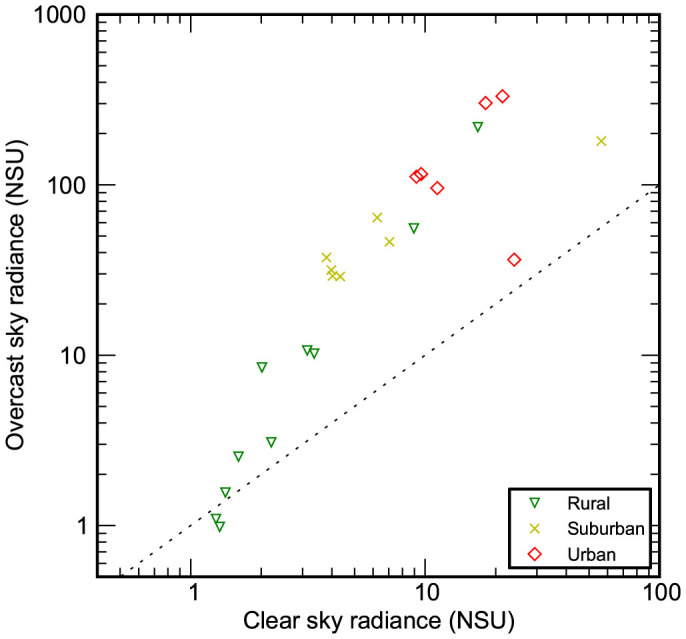
Comparison of clear to overcast sky radiance. The relationship between median midnight clear and overcast sky radiance is shown for locations at which cloud coverage data were available. A dashed 1:1 line is shown for reference. Points above the line are areas where clouds make the sky brighter, whereas below the line clouds make the sky darker.

**Figure 4:**
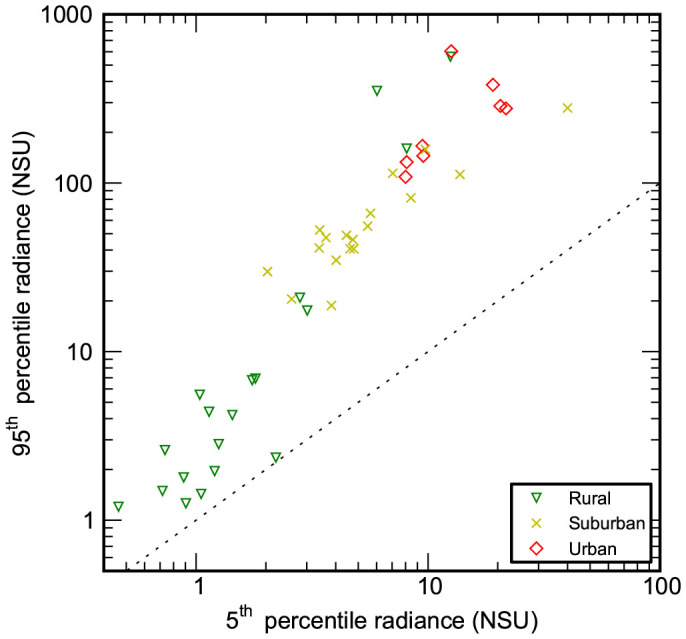
Comparison of 5^th^ to 95^th^ percentile in sky brightness. The extremes in sky radiance are shown for all sites at all periods of astronomical night. A dashed 1:1 line is shown for reference; points on this line would have zero variation in sky brightness under all weather conditions. Locations which have 5th percentile values below 1 NSU likely indicate that the sky is darker when overcast.

Based on median midnight sky radiance, overcast skies were brighter than clear skies (Fig. 3, Table S3) at nearly all locations. The variation in skyglow radiance was larger on overcast nights than on clear nights at all sites (Table S5). In order to compare the radiance of clear and cloudy nights at sites lacking cloud coverage data, the 5^th^ and 95^th^ percentile of sky radiance observations for all locations were used (Fig. 4). Sites of similar character were again observed to cluster. The 5^th^ percentile value is darker than the expected clear sky signal at a number of sites (NSU < 1), making it very likely that clouds darken the night sky at these sites.

The “brightening factor” (ratio of overcast to clear sky radiance) was calculated for each site, and tended to increase along a rural → urban gradient of increasing clear sky radiance (Table S3). There was a relationship between overcast and clear sky radiance, but it was not of the form o = *a*c*^k^* (where o and c represent overcast and clear sky radiance). Instead, the relationship appears to be curved, with an inflection around 2–4 times the natural night sky brightness. The sites below this point are all rural or pristine. This suggests that the change in the relationship may be related to a transition from sites for which the lights from a distant city could be approximated as originating from a point source, to sites located nearer or within city limits.

Kocifaj and Solano Lamphar^42^ recently performed radiative transfer simulations to examine the relationship between the cloud brightening factor for irradiance and distance from a city center. They predicted that brightening factors increase rapidly as cities are approached, but are likely to decrease as the city boundary is crossed. In contrast, the largest brightening factors observed in the dataset were found near the centers of Plymouth, UK (17.6) and Berlin, Germany (16.2). While the differences in experimental design prevent a direct comparison, it is notable that an inflection occurs at the city boundary in both the observational and simulated data. These data allow a connection between theoretical and observational studies on large spatial scales for the first time, and future cooperation between experimentalists and theorists will allow rigorous testing of skyglow models.

### Temporal change in sky radiance

**Figure 5:**
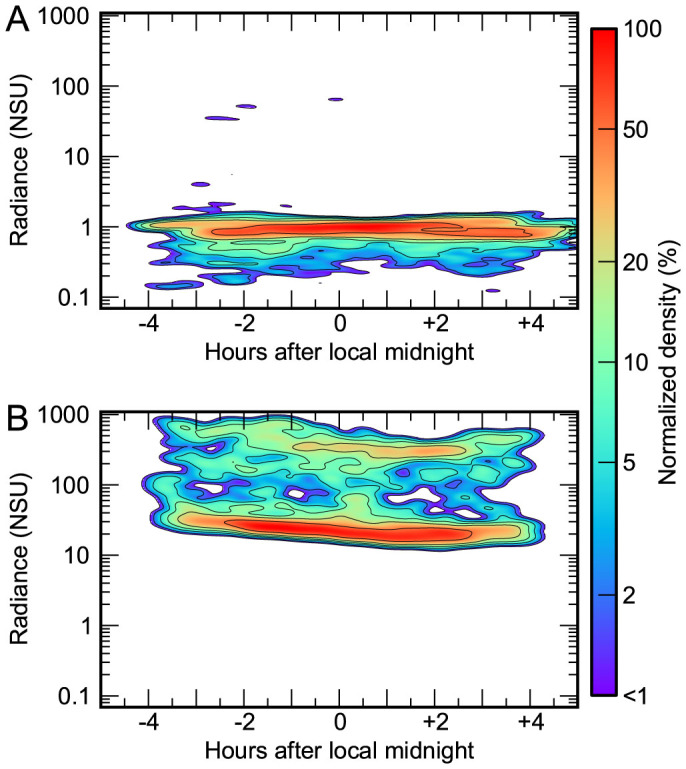
Contour plot showing observed sky brightness during moonless nights over the full data period. Panel A shows Kitt Peak, AZ, USA (5283 observations on 94 nights), Panel B shows Hackescher Markt, Berlin, Germany (1061 observations on 44 nights). For reference, 1000 NSU is 14.1 mag*_SQM_*/arcsec^2^, and 10 NSU is 19.1 mag*_SQM_*/arcsec^2^. Panel B also displays the separation into two typical regimes corresponding to clear and overcast conditions typical of bright sites (c.f. Ref. 37).

In general, areas with little artificial skyglow tend to have little variation therein over the course of the night (Fig. 5A). In contrast, at brighter locations there is usually a visible trend towards decreasing radiance as the night progresses (Fig. 5B). At sites dominated by artificial light (NSU > 2), the median decrease in the artificial component of skyglow was found to be about ~5% per hour, both before and after midnight (Fig. S9 and Fig. S10).

## Discussion

This study has documented the remarkable extent to which the Earth's environment has been changed by the addition of artificial light. In this era of historically unprecedented light levels, lunar cycles are masked, clouds brighten the environment rather than darken it, and the early and late night are no longer physically equivalent. These changes mean that some light dependent processes that take place during crepuscular or moonlit periods (e.g. visual hunting) will be possible at night. Conversely, the darkness that was associated with cloudy nights before the anthropocene is no longer available as a resource (e.g. for adult insects emerging from water): only about one third of the sites experienced regular periods in which the sky was less than twice as bright as a natural starlit sky.

These observations are the most detailed study of night sky brightness ever reported, and yet represent just a snapshot of Earth's physical environment during a period of rapid change in artificial lighting. Street lighting is in a particularly dynamic phase due to technological development, budgetary demands, energy scarcity, and desired transition to more sustainable cities^43^,^44^,^45^. The establishment of an international network of sky brightness monitoring sites should be a high priority. In addition to improving understanding of this global environmental change, the data from such a network are crucially needed to test and improve the increasingly sophisticated skyglow models under development^46^,^47^,^48^. These models can then be used to interpolate into regions where monitoring is not taking place, which will greatly aid understanding of the diverse social and environmental costs of skyglow.

## Methods

### Measurement device and units

The SQM measures sky radiance in a cone of about 20° (full width at half maximum) in a spectral band that is similar, but not identical to, the visual band for which luminance is defined^49^,^50^. Measurements are taken in the logarithmic astronomical units of mag/arcsec^2^. The mag/arcsec^2^ scale is constructed so that a *decrease* of 5 in mag/arcsec^2^ corresponds to a factor of 100 *increase* in radiance. We follow the convention of Biggs^32^ and other authors and report all measurements in terms of the SQM spectral band mag*_SQM_*/arcsec^2^. An approximate conversion to luminance is possible using the formula cd/m^2^ = 10.8 × 10^4^ × 10^−0.4*x*^, where *x* is the radiance in mag*_SQM_*/arcsec^2^. However, at artificially lit locations, the sky becomes redder with increasing cloud cover^33^,^42^, and this conversion would likely overestimate the luminance.

Since the mag/arcsec^2^ scale is not familiar to most readers, in many places the results are reported in “natural sky units” (NSU). A value in NSU indicates how much brighter or darker the sky is compared to a typical historic clear night sky. It is defined here as NSU = 10^0.4Δ^, where Δ is 21.6 minus the observed value in mag*_SQM_*/arcsec^2^.

### Observation locations

Sky radiance data were collected by professional researchers and citizen scientists using 54 SQMs at 50 locations (in some cases the SQM was swapped or used in multiple locations). The observing sites were located in the USA (12), Netherlands (9), Germany (8), Italy (5), Canada (4), UK (3), Australia (2), Austria (2), Spain (2), Japan (1), Mexico (1), and Norway (1) (Tab. S1, Tab. S2). Data contributors classified their site as “urban”, “suburban”, “rural” (within 100 km of a city of 50,000 or more), or “pristine” (far from cities and almost no lighting within 50 km). While the locations sample a wide variety of artificial light regimes, from the entirely natural to the entirely urban, they are neither a random nor a representative sample of locations on Earth, and are almost exclusively located in developed countries.

Data were collected primarily in two periods, from 1 May 2011 to 30 September 2011, and 1 May 2012 to 30 September 2012, to avoid the influence of reduced foliage, and frost or snow on the observations. Some SQMs were installed or de-installed during the measurement period (e.g. to avoid monsoon seasons), and uptime was sometimes reduced due to problems such as readout computer or power failures (Tab. S2). Data from two sites in Australia were taken between 21 and 29 November 2011 (Alice Springs) and from 15 March to 29 April 2012 and 21 May to 1 October 2012 (Adelaide). Some subsamples of these data have been reported previously^29^,^30^,^33^,^35^,^37^,^38^, but this is the first time the datasets have been systematically contrasted with each other.

All devices were installed in a weatherproof housing and aimed at zenith. Results were corrected for the extinction coefficient; two sites are excluded from the analyses because it was unknown (Tab. S3). The manufacturer reports that unit-to-unit differences between SQMs result in a systematic uncertainty of 0.1 mag*_SQM_*/arcsec^2^ (~10% in luminance), consistent with the differences observed in field campaigns^51^. Data were taken using SQM-LE, SQM-LU, and SQM-LU-DL devices. The devices are optically identical, and differ in how they are read out. The Ethernet connector of the SQM-LE produces some internal heating, but the light sensor has a known temperature dependence that is internally corrected before readout^52^.

### Data processing

Data were taken using a variety of different file formats, with different time references (e.g. UTC, local, and unix time). These were converted to a uniform format, and each group verified that the time was properly encoded for their site. To improve future data exchange, a standard format for reporting skyglow measurement was developed in consultation with skyglow researchers worldwide. The standard was officially adopted on 15 September 2012 at the 12th European Symposium for the Protection of the Night Sky^53^.

The sampling rate at the sites ranged from a minimum of 1 observation every 15 minutes to a maximum of 1 observation per second. To simplify the analysis, data from sites with sampling rates greater than one observation per minute were averaged to produce a minute-by-minute dataset. Two locations were affected by a software thresholding problem, in which data were not recorded when the sky was darker than 20 mag*_SQM_*/arcsec^2^ (Tab. S3), and were not used in the analyses. Four additional locations were rejected from the analysis because they experienced SQM or setup failures that resulted in inconsistent data (Tab. S3). As a result, the total number of observing sites was reduced from 50 to 44.

The total amount of data from each site varied due to the sampling rate, the period over which the SQM was installed and working, and latitude. Data were rejected if the sun was not more than 18° below the horizon (astronomical night). With the exception of the moonlight cloudy night analysis, periods during which the moon was above the horizon were also rejected. To separate the effects of clouds and temporal changes in skyglow, some analyses restrict data to periods near to “midnight”. Here, midnight is defined as the hour that falls closest to the time when the sun reaches its deepest point below the horizon for each individual site. Depending on the observation's location relative to a time zone boundary and whether a community uses daylight savings time, “midnight” could be 23:00, 00:00, 01:00, or 02:00 in local time (e.g. in Berlin, “midnight” occurs at 01:00 local time).

### Cloud coverage analysis

The analysis follows a method similar to that used by Kyba et al.^29^,^33^. Cloud coverage was obtained from SYNOP reports downloaded from the ogimet website for the SYNOP station nearest to the site (www.ogimet.com). This distance ranged from 3 to 112 km. SYNOP reports describe fractional cloud coverage in oktas, and only completely overcast (8 oktas) and completely clear (0 okta) conditions are considered. The clear and overcast sky radiances are defined as the median radiance observed within ±15 minutes of midnight under the given cloud condition.

Approximately half of the SYNOP stations did not provide hourly reports, so the cloud coverage analysis was not possible for these sites. To extend this analysis to include data from all sites, the relationship between brightness percentile and clear sky radiance at sites with SYNOP data was investigated. The 28th percentile was found to match the clear sky radiances the best, and the 81st percentile was found to match overcast sky radiances best. We also compared the 5th percentile in observed sky radiance (darkest nights) to the 95th percentile. For urban sites the 5th percentile occurs on clear nights, whereas for pristine locations, the 5th percentile likely occurs on overcast nights.

### Comparison to World Atlas

The median sky radiance observed on cloud free nights was compared to the predictions of the “First World Atlas of Artificial Night Sky Brightness”^20^ for sites in Europe and North America. The georeferencing of the Atlas was known to be off by about a pixel, so it was newly georeferenced. Whereas the Atlas was calculated for nights with a fairly transparent atmosphere, nights with clear skies but high humidity or aerosol content would be included in our analysis. Additionally, the estimates in the World Atlas are for the Johnson V band, which is not the same as V_SQM_. Finally, the satellite data for some of the Northern latitude sites was mainly taken during winter periods.

### Temporal radiance change analysis

Temporal change was studied in two ways. First, contour plots showing all of the moon-free night data were produced for each site (this technique was first published in Ref. 37, and was also independently presented earlier at workshops by den Outer.) Contours were calculated using Gaussian kernel estimation, and can be visually inspected for trends. Second, the sites found to be primarily artificially lit (20.85 mag*_SQM_*/arcsec^2^ or brighter) were studied to find the rate of change in the artificial light component. The median observed clear sky radiance was found for these sites at intervals of ±15 minutes around each of 22:00, 00:00, and 02:00 (where 00:00 is “midnight” as described above). Radiances were converted to NSU, and the assumed natural background of 1 NSU was subtracted. The rate of change over each two hour interval was then calculated.

### Overcast moonlit night analysis

The SQM is designed to measure a relatively uniformly lit field, and point-like sources such as the moon do not match this assumption. However, on completely overcast nights, the radiance of moonlight leaving the cloud base can be assumed to have little zenith dependence (similar to the overcast sky in daytime, see e.g. Ref. 54.) Contour plots of overcast sky radiance against moon elevation were produced for sites with SYNOP data (Fig. S6). To minimize any effect from temporal changes, only data taken within 15 minutes of midnight were considered. Note that this timing restriction introduces a relationship between lunar elevation and phase. Plots were only produced for sites with at least 40 data points, and at least one observation taken on a moonlit night.

## Supplementary Material

Supplementary Information Supplementary Information

## Figures and Tables

**Figure 1:**
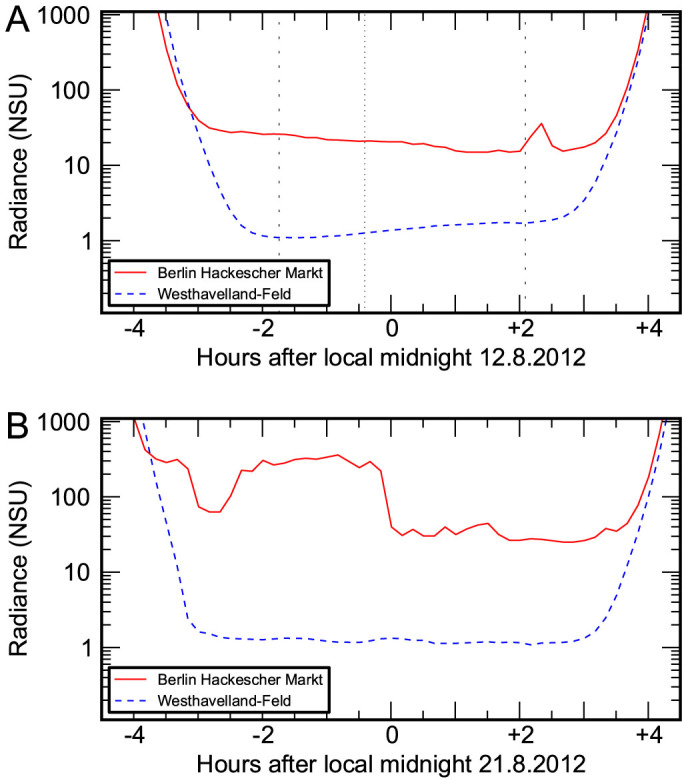
Comparison of scotographs for urban and rural locations. Panel A shows the sky radiance in “natural sky units” (relative to an assumed natural radiance of 21.6 mag*_SQM_*/arcsec^2^, see methods) for a clear night in a city center (solid red) and nearby nature reserve (dashed blue). The sky radiance was similar until shortly before astronomical night began (dashed vertical lines). The sky in the reserve grew brighter as the 36% illuminated moon rose (dotted vertical line), but the sky in the city grew darker. Panel B shows scotographs taken on a cloudy night. In the city, sky radiance changed by more than an order of magnitude as clouds passed over, while the response was more muted in the country.

## References

[b1] Central Intelligence Agency. The World Factbook. URL: https://www.cia.gov/library/publications/the-world-factbook/fields/2085.html (2013). Access date 25 August, 2014.

[b2] Longcore T. & Rich C. Ecological light pollution. Front Ecol Environ 2, 191–198 (2004).

[b3] Rich, C. & Longcore, T. (eds.) Ecological Consequences of Artificial Night Lighting (Island Press, Washington, D.C., USA, 2006).

[b4] Hölker F., Wolter C., Perkin E. K. & Tockner K. Light pollution as a biodiversity threat. Trends Ecol Evol 25, 681–682 (2010). 21035893 10.1016/j.tree.2010.09.007

[b5] Perkin E. *et al.* The influence of artificial light on freshwater and riparian ecosystems: Questions, challenges, and perspectives. Ecosphere 2, art122 (2011).

[b6] Gaston K. J., Bennie J., Davies T. W. & Hopkins J. The ecological impacts of nighttime light pollution: a mechanistic appraisal. Biological Reviews 88, 912–927 (2013). 23565807 10.1111/brv.12036

[b7] Navara K. J. & Nelson R. J. The dark side of light at night: physiological, epidemiological, and ecological consequences. J Pineal Res 43, 215–224 (2007). 17803517 10.1111/j.1600-079X.2007.00473.x

[b8] Stevens R. G., Brainard G. C., Blask D. E., Lockley S. W. & Motta M. E. Adverse health effects of nighttime lighting: comments on american medical association policy statement. American journal of preventive medicine 45, 343–346 (2013). 23953362 10.1016/j.amepre.2013.04.011

[b9] Fonken L. K. & Nelson R. J. The effects of light at night on circadian clocks and metabolism. Endocrine reviews 35, 648–670 (2014). 24673196 10.1210/er.2013-1051

[b10] Stevens R. G., Brainard G. C., Blask D. E., Lockley S. W. & Motta M. E. Breast cancer and circadian disruption from electric lighting in the modern world. CA: a cancer journal for clinicians 64, 207–218 (2014). 24604162 10.3322/caac.21218 PMC4038658

[b11] Davies T. W., Bennie J. & Gaston K. J. Street lighting changes the composition of invertebrate communities. Biology letters rsbl20120216 10.1098/rsbl.2012.0216 (2012). 10.1098/rsbl.2012.0216 PMC3440964 22628095

[b12] Nordt A. & Klenke R. Sleepless in town–drivers of the temporal shift in dawn song in urban European blackbirds. PLOS ONE 8, e71476 (2013). 23940759 10.1371/journal.pone.0071476 PMC3737108

[b13] Dominoni D. M., Carmona-Wagner E. O., Hofmann M., Kranstauber B. & Partecke J. Individual-based measurements of light intensity provide new insights into the effects of artificial light at night on daily rhythms of urban-dwelling songbirds. J Anim Ecol 83, 681–692 (2014). 24102250 10.1111/1365-2656.12150

[b14] Lewanzik D. & Voigt C. C. Artificial light puts ecosystem services of frugivorous bats at risk. J Appl Ecol 51, 388–394 (2014).

[b15] Lucas R. J. *et al.* Measuring and using light in the melanopsin age. Trends Neurosci 37, 1–9 (2014). 24287308 10.1016/j.tins.2013.10.004 PMC4699304

[b16] Perkin E. K., Hölker F. & Tockner K. The effects of artificial lighting on adult aquatic and terrestrial insects. Freshwater Biology 59, 368–377 (2014).

[b17] Kyba C. C. M. & Hölker F. Do artificially illuminated skies affect biodiversity in nocturnal landscapes? Landscape Ecol 28, 1637–1640 (2013).

[b18] Cinzano P., Falchi F. & Elvidge C. D. Naked-eye star visibility and limiting magnitude mapped from DMSP-OLS satellite data. Mon Not R Astron Soc 323, 34–46 (2001).

[b19] Crumey A. Human contrast threshold and astronomical visibility. Mon Not R Astron Soc 442, 2600–2619 (2014).

[b20] Cinzano P., Falchi F. & Elvidge C. D. The first World Atlas of the artificial night sky brightness. Mon Not R Astron Soc 328, 689–707 (2001).

[b21] Kyba C. C. M., Ruhtz T., Fischer J. & Hölker F. Lunar skylight polarization signal polluted by urban lighting. J Geophys Res 116, D24106 (2011).

[b22] Kronfeld-Schor N. & Dayan T. Partitioning of time as an ecological resource. Annu Rev Ecol Evol S 34, 153–181 (2003).

[b23] Dacke M., Nilsson D.-E., Scholtz C. H., Byrne M. & Warrant E. J. Insect orientation to polarized moonlight. Nature 424, 33 (2003). 10.1038/424033a 12840748

[b24] Dacke M., Baird E., Byrne M., Scholtz C. & Warrant E. Dung beetles use the milky way for orientation. Curr Biol 23, 298–300 (2013). 23352694 10.1016/j.cub.2012.12.034

[b25] Clarke J. A. Moonlight's influence on predator/prey interactions between short-eared owls (*asio flammeus*) and deermice (*peromyscus maniculatus*). Behav Ecol Sociobiol 13, 205–209 (1983). 10.1007/BF00299924.

[b26] Moore M. V., Pierce S. M., Walsh H. M., Kvalvik S. K. & Lim J. D. Urban light pollution alters the diel vertical migration of *daphnia*. Verh. Internat. Verein. Limnol. 27, 779–782 (2000).

[b27] Gallaway T. On light pollution, passive pleasures, and the instrumental value of beauty. J Econ Issues 44, 71–88 (2010).

[b28] Riegel K. Light pollution. Science 179, 1285–1291 (1973). 17835929 10.1126/science.179.4080.1285

[b29] Kyba C. C. M., Ruhtz T., Fischer J. & Hölker F. Cloud coverage acts as an amplifier for ecological light pollution in urban ecosystems. PLOS ONE 6, e17307 (2011). 21399694 10.1371/journal.pone.0017307 PMC3047560

[b30] Lolkema D., Haaima M., den Outer P. & Spoelstra H. Effects of meteorological and atmospheric parameters on night sky brightness. Tech. Rep. RIVM #680151002, Netherlands National Institute for Public Health and the Environment, Bilthoven, Netherlands (2011). Available at: http://rivm.openrepository.com/rivm/handle/10029/262205 (Accessed: 4th November 2014).

[b31] Pun C. & So C. Night-sky brightness monitoring in Hong Kong: a city-wide light pollution assessment. Environ Monit Assess 184, 2537–2557 (2012). 21713499 10.1007/s10661-011-2136-1

[b32] Biggs J. D., Fouch T., Bilki F. & Zadnik M. G. Measuring and mapping the night sky brightness of Perth, Western Australia. Mon Not R Astron Soc 421, 1450–1464 (2012).

[b33] Kyba C. C. M., Ruhtz T., Fischer J. & Hölker F. Red is the new black: How the color of urban skyglow varies with cloud cover. Mon Not R Astron Soc 425, 701–708 (2012).

[b34] Ścięz·or T., Kubala M. & Kaszowski W. Light pollution of the mountain areas in Poland. Arch Environ Prot 38, 59–69 (2012).

[b35] Davies T. W., Bennie J., Inger R. & Gaston K. J. Artificial light alters natural regimes of night-time sky brightness. Sci Rep 3, 1772 (2013).

[b36] Kyba C. C. M. *et al.* Citizen science provides valuable data for monitoring global night sky luminance. Sci Rep 3, 1835 (2013). 23677222 10.1038/srep01835 PMC3655480

[b37] Puschnig J., Posch T. & Uttenthaler S. Night sky photometry and spectroscopy performed at the Vienna University observatory. J Quant Spectrosc Ra 139, 64–75 (2014).

[b38] Puschnig J., Schwope A., Posch T. & Schwarz R. The night sky brightness at Potsdam-Babelsberg including overcast and moonlit conditions. J Quant Spectrosc Ra 139, 76–81 (2014).

[b39] Pun C., So C., Leung W. & Wong C. Contributions of artificial lighting sources on light pollution in hong kong measured through a night sky brightness monitoring network. J Quant Spectrosc Ra 139, 90–108 (2014).

[b40] Ściężor T. & Kubala M. Particulate matter as an amplifier for astronomical light pollution. Mon Not R Astron Soc 444, 2487–2493 (2014).

[b41] Garstang R. Brightness of clouds at night over a city. The Observatory 127, 1–13 (2007).

[b42] Kocifaj M. & Lamphar H. A. S. Quantitative analysis of night skyglow amplification under cloudy conditions. Mon Not R Astron Soc 443, 3665–3674 (2014).

[b43] Falchi F., Cinzano P., Elvidge C., Keith D. & Haim A. Limiting the impact of light pollution on human health, environment and stellar visibility. J Environ Manage 92, 2714–2722 (2011). 21745709 10.1016/j.jenvman.2011.06.029

[b44] Gaston K. J. Sustainability: A green light for efficiency. Nature 497, 560–561 (2013). 23719447 10.1038/497560a

[b45] Kyba C., Hänel A. & Hölker F. Redefining efficiency for outdoor lighting. Energ Environ Sci 7, 1806–1809 (2014).

[b46] Aubé M. & Kocifaj M. Using two light-pollution models to investigate artificial sky radiances at canary islands observatories. Mon Not R Astron Soc 422, 819–830 (2012).

[b47] Cinzano P. & Falchi F. The propagation of light pollution in the atmosphere. Mon Not R Astron Soc 427, 3337–3357 (2012).

[b48] Kocifaj M. Modeling the night-sky radiances and inversion of multi-angle and multi-spectral radiance data. J Quant Spectrosc Ra 139, 35–42 (2014).

[b49] Cinzano P. Night sky photometry with Sky Quality Meter. Tech. Rep. 9, ISTIL (2005). V1.4. Available at: http://www.lightpollution.it/download/sqmreport.pdf (Accessed:14th January 2015).

[b50] Cinzano P. Report on Sky Quality Meter, version L. Tech. Rep. ISTIL (2007). Available at: http://unihedron.com/projects/sqm-l/sqmreport2.pdf (Accessed:14th January 2015).

[b51] den Outer P. *et al.* Intercomparisons of nine sky brightness detectors. Sensors 11, 9603–9612 (2011). 22163715 10.3390/s111009603 PMC3231263

[b52] Schnitt S., Ruhtz T., Fischer J., Hölker F. & Kyba C. Temperature stability of the sky quality meter. Sensors 13, 12166–12174 (2013). 24030682 10.3390/s130912166 PMC3821345

[b53] Kyba C. & Lolkema D. A community standard for recording skyglow data. Astron Geophys 53, 6–17 (2012).

[b54] Petty G. W. A first course in atmospheric radiation (Sundog Publishing, 2006).

