# Supplementary material for: Worldwide variations in artificial skyglow
Source: Sci Rep. 2015 Feb 12;5:8409. doi: 10.1038/srep08409 (PMC5389131; doi:10.1038/srep08409)

Supplemental Information

Worldwide variations in artificial skyglow

Christopher C. M. Kyba[1,2,3,*], Kai Pong Tong[4], Jonathan Bennie[5], Ignacio Birriel[6], Jennifer J. Birriel[6], Andrew Cool[7], Arne Danielsen[8], Thomas W. Davies[5], Peter N. den Outer[9], William Edwards[8], Rainer Ehlert[8], Fabio Falchi[10], Jürgen Fischer[2], Andrea Giacomelli[11], Francesco Giubbilini[11], Marty Haaima[9], Claudia Hesse[11], Georg Heygster[4], Franz Hölker[1], Richard Inger[5], Linsey J. Jensen[13,14], Helga U. Kuechly[1], John Kuehn[8], Phil Langill[15], Dorien E. Lolkema[9], Matthew Nagy[16], Miguel Nievas[17], Nobuaki Ochi[18], Emil Popow[19], Thomas Posch[20], Johannes Puschnig[20], Thomas Ruhtz[2], Wim Schmidt[21], Robert Schwarz[19], Axel Schwope[19], Henk Spoelstra[22], Anthony Tekatch[23], Mark Trueblood[24], Constance E. Walker[13], Michael Weber[19], Douglas L. Welch[25], Jaime Zamorano[17], Kevin J. Gaston[5]

[1] **Leibniz Institute for Freshwater Ecology and Inland Fisheries**
[2] **Institute for Space Sciences, Freie Universität Berlin, Berlin, Germany**
[3] **Deutsches GeoForschungsZentrum Potsdam, Potsdam, Germany**
[4] **Institut für Umweltphysik, Universität Bremen, Bremen, Germany**
[5] **Environment and Sustainability Institute, University of Exeter, Penryn, UK**
[6] **Morehead State University, Morehead, USA**
[7] **The Heights Observatory, Adelaide**
[8] **Unaffiliated Citizen Scientist**
[9] **National Institute for Public Health and the Environment, Bilthoven, The Netherlands**
[10] **Light Pollution Science and Technology Institute (ISTIL), Thiene, Italy**
[11] **Attivarti.org, Torniella, Italy**
[12] **Landesamt für Umwelt, Gesundheit und Verbraucherschutz Brandenburg, Germany**
[13] **National Optical Astronomy Observatory, Tucson, USA**
[14] **Utah State University, Logan, USA**
[15] **Rothney Astrophysical Observatory, University of Calgary, Calgary, Canada**
[16] **Department of Physics, University of Alberta, Edmonton, Canada**
[17] **Departamento de Astrofísica y Ciencias de la Atmósfera, Universidad Complutense de Madrid, Madrid, Spain**
[18] **Faculty of Business Administration, Toyo University, Tokyo, Japan**
[19] **Leibniz Institut für Astrophysik Potsdam (AIP), Potsdam, Germany**
[20] **Institut für Astrophysik, Vienna, Austria**
[21] **Sotto le Stelle, Utrecht, The Netherlands**
[22] **Lumineux Consult, Arnhem, The Netherlands**
[23] **Unihedron, Grimsby, Canada**
[24] **Winer Observatory, Sonoita, USA**
[25] **Department of Physics and Astronomy, McMaster University, Hamilton, Canada**
∗ **E-mail: kyba@gfz-potsdam.de**

Page 3: Supplemental Table S1 shows the location information for the 50 sites. In two cases (Deerlick and Steglitz, Berlin), sky brightness was measured by two SQMs. The sites are ordered by increasing observed sky brightness.

Page 4: Supplemental Table S2 shows the detailed information about the sites, including their physical location (latitude, longitude, and elevation), as well as information about the instrument uptime. Table columns 2011 and 2012 indicate the percentage of time during the study period for which the instruments were online. The column labeled "Up (%)" shows the ratio between the time online and the intended time online (e.g. Site 1 was only installed midway through the second measurement period, and consistently delivered data through this period).

Page 5: Supplemental Table S3 shows the summary of night sky observations during the measurement period. The column "data points" shows how many minute-by-minute observations were made under conditions of astronomical night within 15 minutes of midnight (as defined in the text). The column "WA" indicates the prediction for the clear sky brightness in mag/arcsec^2 by [20] for sites in North America and Europe. "Clear" and "overcast" indicate the sky radiance (in mag$_{SQM}$/arcsec^2) under these conditions; values marked with a $*$ did not have SYNOP data, and the clear and overcast values are the 28th and 81st percentiles. The "brightening factor" indicates the linear ratio of overcast to clear sky radiance for sites which had SYNOP data. The 22 $\rightarrow$ 24 and 24 $\rightarrow$ 02 columns show the change in clear sky radiance between the given times. Sites 46-52 are not used in the analysis due to instrumental problems (see methods).

Page 6: Supplemental Table S4 shows the difference in overcast sky radiance at midnight (8 okta) nights for nights with the moon below or above the horizon.

Page 7: Supplemental Table S5 shows the variation in sky radiance on clear and cloudy midnights. Only sites with both SYNOP observations and both clear and overcast (8 okta) data are included.

Page 7: Supplemental Table S6 shows the variation in sky radiance on moonlit and moon-free overcast midnights. Only sites with SYNOP data, at least 40 observations, and data on both moonlit and moon-free nights are shown.

Pages 8-11: Supplemental Figure S7 is a composite figure showing the relationship between lunar elevation and sky radiance on overcast nights. Only sites with SYNOP data, at least 40 observations, and data on both moonlit and moon-free nights are shown.

Pages 12-18: Supplemental Figure S8 is a composite figure showing the observed change in sky radiance (in mag$_{SQM}$/arcsec^2) over the course of the night on moon-free nights for each of the 50 sites.

Page 19: Supplemental Figure S9 is a histogram showing the observed change in median sky radiance from 22:00 to midnight on a linear scale. Values larger than unity indicate that a site becomes brighter as the night progresses. Only sites with SYNOP data and midnight clear sky radiances brighter than 20.85 mag$_{SQM}$/arcsec^2 ($\sim$2 NSU) are analyzed.

Page 19: Supplemental Figure S10 is a histogram showing the observed change in median sky radiance from midnight to 02:00 on a linear scale. Values larger than unity indicate that a site becomes brighter as the night progresses. Only sites with SYNOP data and midnight clear sky radiances brighter than 20.85 mag$_{SQM}$/arcsec^2 ($\sim$2 NSU) are analyzed.

Supplemental Table S11 contains the entire dataset, and can be downloaded separately from the publisher's website. The data format is as follows:
Site number: location
Year (UTC time)
Month (UTC time)
Day (UTC time)
Hour (UTC time)
Minute (UTC time)
Hours after local midnight
Solar elevation angle
Lunar elevation angle
Number of observations in 1-minute interval
Observed radiance in mag$_{SQM}$/arcsec^2 (Corrected for window attenuation)

Supplemental Table S1: Site designations

N	Site name	Description	Loc
1	Tucson KP	Kitt Peak, Tucson, AZ	US
2	Schiermnk	Schiermonnikoog	NL
3	Veinto Rg	Veinto Ridge, Silver City, NM	US
4	Tucson Hpk	Mount Hopkins, Tucson AZ	US
5	Winer	Winer Observatory, Sonoita, AZ	US
6	Alice Sp	Alice Springs	AU
7	IP Feld	Westhavelland, Brandenburg	DE
8	Tucson Lm	Mount Lemmon, Tucson, AZ	US
9	Parey	Parey, Brandenburg	DE
10	Tenerife	STELLA Robotic Observatory, Izana, Tenerife	ES
11	Springendal	Springendal, Hezingen	NL
12	Rad Kwk	Radio Kootwijk	NL
13	Seebergen	Seebergen, Lilienthal	DE
14	Tucson W	Tucson West, AZ	US
15	Calgary RAO	Rothney Astrophysical Observatory Calgary	CA
16	Vitto Veneto	Central Vittorio Veneto	IT
17	Arkemheen	Arkemheen	NL
18	CESAR	CESAR, Cabauw	NL
19	Potsdam Bb	Babelsberg, Potsdam	DE
20	Arnhem	Arnhem	NL
21	Vestby	Vestby	NO
22	Lendinara	Lendinara	IT
23	Berlin Sd	Schulzendorf, Berlin	DE
24	Tucson N	Tucson North, AZ	US
25	Grimsby	Grimsby, ON	CA
26	Met Monte	MeteoMontebello	IT
27	Castigli	Castiglioncello	IT
28	Nove	Nove	IT
29	Bremen H-L	Horn-Lehe, Bremen	DE
30	Morehead	Morehead, KY	US
31	Hamilton	Hamilton, ON	CA
32	Berlin St LE	Steglitz, Berlin (SQM-LE)	DE
33	Berlin St LU	Steglitz, Berlin (SQM-LU)	DE
34	Adelaide	Adelaide	AU
35	Tucson E	Tucson East, AZ	US
36	Tucson C	Tucson Center, AZ	US
37	Utrecht	Utrecht	NL
38	Vienna IFA	Institute of Astronomy, University of Vienna	AT
39	Schipluiden	Schipluiden	NL
40	Plymouth Dv	Plymouth University, Plymouth	UK
41	Tucson S	Tucson South, AZ	US
42	Berlin HM	Hackescher Markt, Berlin	DE
43	Madrid UCM	Universidad Complutense de Madrid, Madrid	ES
44	Edmonton	Edmonton, AB	CA
45	Vlaardingen	Vlaardingen	NL
46	Yonago[¶]	Yonago	JP
47	Mexico[¶]	San Luis Potosì	MX
48	Plymouth Rm[†]	Ramehead, Torpoint, Cornwall	UK
49	Plymouth Mk[†]	Looe, Cornwall	UK
50	FOA Schöpfl[‡]	Leopold-Figl-Observatorium für Astrophysik, Mt. Mitterschöpfl	AT
51	Deerlick 1[§]	Deerlick Astronomical Village, Crawfordville, GA (Station 1)	US
52	Deerlick 2[§]	Deerlick Astronomical Village, Crawfordville, GA (Station 2)	US

[¶] Window attenuation unknown
[†] Thresholding problem
[‡] Instrument failure under dark skies.
[§] Instrument failure

Supplemental Table S2: Detailed site information

N	Site name	Loc	Class	Lat (°N)	Lon (°E)	Elev (m)	2011	2012	Up (%)
1	Tucson KP	US	rural	31.96	-111.60	2065	N/A	74.4	99.3
2	Schiermnk	NL	rural	53.48	6.20	7	37.3	93.0	67.7
3	Veinto Rg	US	rural	32.77	-108.33	1936	81.7	N/A	81.7
4	Tucson Hpk	US	rural	31.68	-110.88	2335	N/A	68.0	88.7
5	Winer	US	rural	31.67	-110.60	1516	27.7	15.5	30.8
6	Alice Sp	AU	rural	-23.50	133.84	718	99.8	N/A	99.8
7	IP Feld	DE	rural	52.70	12.46	27	N/A	91.0	91.0
8	Tucson Lm	US	rural	32.44	-110.79	2781	N/A	66.4	89.1
9	Parey	DE	rural	52.68	12.25	36	N/A	82.4	82.4
10	Tenerife	ES	rural	28.30	-16.51	2380	N/A	91.4	91.4
11	Springendal	NL	rural	52.43	6.88	74	80.8	90.3	90.0
12	Rad Kwk	NL	rural	52.11	5.49	71	67.0	27.3	49.7
13	Seebergen	DE	rural	53.14	8.99	11	N/A	86.3	91.8
14	Tucson W	US	suburban	32.24	-111.08	856	N/A	65.6	85.5
15	Calgary RAO	CA	rural	50.87	-114.29	1269	N/A	19.0	19.0
16	Vitto Veneto	IT	suburban	46.01	12.31	500	6.2	34.0	20.1
17	Arkemheen	NL	rural	52.25	5.43	4	75.6	89.8	87.2
18	CESAR	NL	rural	51.97	4.93	3	94.5	73.2	88.7
19	Potsdam Bb	DE	suburban	52.40	13.10	66	97.9	100.0	99.0
20	Arnhem	NL	suburban	51.94	5.87	14	93.4	88.9	96.5
21	Vestby	NO	suburban	59.60	10.76	84	96.7	99.8	66.5
22	Lendinara	IT	suburban	45.08	11.56	9	78.4	7.3	42.8
23	Berlin Sd	DE	suburban	52.36	13.59	50	N/A	90.1	90.1
24	Tucson N	US	suburban	32.32	-111.89	863	N/A	22.2	28.7
25	Grimsby	CA	suburban	43.18	-79.52	173	98.7	99.0	98.9
26	Met Monte	IT	suburban	45.47	11.35	212	72.7	96.1	84.4
27	Castigli	IT	suburban	43.41	10.42	15	85.1	N/A	85.1
28	Nove	IT	suburban	45.71	11.68	87	56.0	94.1	75.1
29	Bremen H-L	DE	suburban	53.10	8.85	22	N/A	79.9	79.9
30	Morehead	US	rural	38.19	-83.44	243	11.0	55.0	11.0
31	Hamilton	CA	suburban	43.27	-79.96	99	98.6	N/A	98.6
32	Berlin St LE	DE	urban	52.46	13.31	91	81.7	1.4	42.7
33	Berlin St LU	DE	urban	52.46	13.31	77	91.4	98.7	98.1
34	Adelaide	AU	rural	-34.62	138.46	25	N/A	40.6	40.6
35	Tucson E	US	suburban	32.23	-110.80	796	N/A	23.4	29.9
36	Tucson C	US	urban	32.23	-110.95	745	N/A	66.8	84.6
37	Utrecht	NL	urban	52.08	5.11	6	69.0	89.3	54.6
38	Vienna IFA	AT	suburban	48.23	16.33	240	N/A	95.6	95.6
39	Schipluiden	NL	rural	51.99	4.28	1	88.3	87.4	92.9
40	Plymouth Dv	UK	urban	50.37	-4.14	36	N/A	98.1	98.1
41	Tucson S	US	suburban	32.11	-110.96	784	N/A	27.0	42.8
42	Berlin HM	DE	urban	52.52	13.40	46	N/A	90.0	90.0
43	Madrid UCM	ES	urban	40.45	-3.73	650	N/A	46.5	46.5
44	Edmonton	CA	urban	53.53	-113.53	695	N/A	53.6	53.6
45	Vlaardingen	NL	suburban	51.91	4.32	4	61.7	88.5	79.6
46	Yonago¶	JP	suburban	35.46	133.29	24	49.8	N/A	49.8
47	Mexico¶	MX	urban	22.14	-101.03	1950	93.6	N/A	93.6
48	Plymouth Rm†	UK	rural	50.32	-4.22	97	N/A	68.3	68.3
49	Plymouth Mk†	UK	rural	50.37	-4.41	134	N/A	50.8	50.8
50	FOA Schöpfl‡	AT	rural	48.08	15.92	880	N/A	100.0	100.0
51	Deerlick 1§	US	pristine	33.56	-82.76	192	39.6	N/A	39.6
52	Deerlick 2§	US	pristine	33.56	-82.76	192	59.5	90.9	75.2

¶ Window attenuation unknown
† Thresholding problem
‡ Instrument failure under dark skies.
§ Instrument failure

Supplemental Table S3: Sky brightness observations

N	Site name	Data points	WA	Clear	Overcast	Brightening factor	22 → 24	24 → 02
1	Tucson KP	358	21.4	21.8∗	21.5∗	N/A	N/A	N/A
2	Schiermnk	1053	21.3	21.3	21.6	0.7	0.05	0.11
3	Veinto Rg	131	21.1	21.7∗	20.9∗	N/A	N/A	N/A
4	Tucson Hpk	352	21.3	21.5∗	21.2∗	N/A	N/A	N/A
5	Winer	1022	21.4	21.6∗	21.4∗	N/A	N/A	N/A
6	Alice Sp	240	N/A	21.5∗	21.5∗	N/A	N/A	N/A
7	IP Feld	91	21.3	21.3	21.5	0.9	-0.03	-0.01
8	Tucson Lm	347	20.8	21.3∗	20.5∗	N/A	N/A	N/A
9	Parey	164	21.3	21.2	21.1	1.1	0.09	0.09
10	Tenerife	2265	N/A	21.3∗	20.9∗	N/A	N/A	N/A
11	Springendal	1933	20.4	21.1	20.6	1.6	0.10	0.00
12	Rad Kwk	1076	19.9	20.7	20.4	1.7	0.07	0.08
13	Seebergen	758	20.2	20.8	19.3	7.4	0.35	0.07
14	Tucson W	334	19.5	20.4∗	18.4∗	N/A	N/A	N/A
15	Calgary RAO	5	19.6	20.7∗	20.7∗	N/A	N/A	N/A
16	Vitto Veneto	300	20.3	20.4∗	18.9∗	N/A	N/A	N/A
17	Arkemheen	1963	19.9	20.4	19.0	4.5	0.02	0.08
18	CESAR	2009	19.8	20.3	19.1	3.9	-0.03	-0.06
19	Potsdam Bb	2453	19.6	20.2	17.7	13.0	0.03	0.09
20	Arnhem	2115	19.6	20.1	17.9	9.3	0.03	0.11
21	Vestby	167	19.6	20.1	17.9	10.3	-0.15	0.04
22	Lendinara	453	20.3	20.1∗	19.3∗	N/A	N/A	N/A
23	Berlin Sd	96	19.8	20.0	17.9	8.4	0.04	0.04
24	Tucson N	133	21.4	19.9∗	17.8∗	N/A	N/A	N/A
25	Grimsby	926	19.4	19.8∗	18.2∗	N/A	N/A	N/A
26	Met Monte	839	20.0	19.7∗	18.1∗	N/A	N/A	N/A
27	Castigli	408	20.3	19.8∗	18.6∗	N/A	N/A	N/A
28	Nove	764	20.0	19.5	17.4	7.5	0.10	0.13
29	Bremen H-L	796	19.3	19.6	17.1	12.0	0.57	-0.04
30	Morehead	575	20.1	19.2	17.2	6.8	N/A	N/A
31	Hamilton	2315	18.9	19.2∗	17.0∗	N/A	N/A	N/A
32	Berlin St LE	894	18.9	19.1	16.4	13.3	0.29	0.12
33	Berlin St LU	2258	18.9	19.2	16.5	13.5	0.22	0.08
34	Adelaide	1119	N/A	19.2∗	16.9∗	N/A	N/A	N/A
35	Tucson E	132	18.8	19.2∗	17.6∗	N/A	N/A	N/A
36	Tucson C	342	18.1	18.6∗	16.8∗	N/A	N/A	N/A
37	Utrecht	1136	19.1	19.0	16.6	9.2	0.04	0.11
38	Vienna IFA	2272	18.8	19.0∗	16.6∗	N/A	N/A	N/A
39	Schipluiden	2100	18.2	18.5	15.8	13.8	-0.46	-0.87
40	Plymouth Dv	175	19.5	18.5	15.4	17.6	N/A	N/A
41	Tucson S	130	18.9	18.6∗	17.0∗	N/A	N/A	N/A
42	Berlin HM	93	18.5	18.3	15.3	16.2	0.20	0.03
43	Madrid UCM	832	17.4	18.1	17.7	1.5	0.20	0.05
44	Edmonton	57	17.2	18.1∗	16.3∗	N/A	N/A	N/A
45	Vlaardingen	2030	18.7	17.2	16.0	3.2	0.13	-0.25
46	Yonago¶	217	N/A	19.9∗	18.6∗	N/A	N/A	N/A
47	Mexico¶	434	18.8	18.9∗	16.3∗	N/A	N/A	N/A
48	Plymouth Rm†	91	20.7	19.0	19.1	0.9	N/A	N/A
49	Plymouth Mk†	38	21.1	19.3	18.6	1.9	N/A	N/A
50	FOA Schöpfl‡	2330	21.0	20.6∗	20.3∗	N/A	N/A	N/A
51	Deerlick 1§	214	21.3	21.1	20.8	1.3	N/A	N/A
52	Deerlick 2§	3414	21.3	21.3	21.3	1.0	N/A	N/A

¶ Window attenuation unknown

† Thresholding problem

‡ Instrument failure under dark skies. Recent measurements with a properly functioning SQM give a clear sky radiance of 21.4 $mag_{SQM}/arcsec^2$ and an overcast sky radiance of 20.3 $mag_{SQM}/arcsec^2$.

§ Instrument failure

Supplemental Table S4: Comparison of overcast moonless and moonlit midnight radiances. Unit: $\mathrm{mag}_{SQM}/\mathrm{arcsec}^2$

Site name	Moonless data points	Median	Moonlit data points	Median	Moon difference
Schiermnk	478	21.6	568	18.9	2.7
IP Feld	22	21.5	21	19.2	2.3
Parey	38	21.1	42	19.1	2.0
Springendal	784	20.6	720	18.5	2.1
Rad Kwk	440	20.4	369	18.9	1.5
Arkemheen	841	19.0	722	18.0	1.0
CESAR	591	19.1	680	18.2	0.9
Potsdam Bb	399	17.7	420	17.5	0.2
Arnhem	1005	17.9	665	17.3	0.6
Vestby	21	17.9	46	17.3	0.5
Nove	40	17.4	20	17.2	0.2
Bremen H-L	60	17.1	30	16.2	0.9
Morehead	140	17.2	251	16.2	1.0
Berlin St LE	60	16.4	210	16.6	0.1
Berlin St LU	285	16.5	330	16.4	0.0
Utrecht	351	16.6	422	16.4	0.2
Schipluiden	762	15.8	965	15.6	0.2
Plymouth Dv	52	15.4	37	15.2	0.2
Madrid UCM	30	17.7	90	15.1	2.5
Vlaardingen	702	16.0	786	15.8	0.1
Plymouth Rm	44	19.1	37	18.4	0.7
Plymouth Mk	12	18.6	30	18.2	0.4
Deerlick 1	18	20.8	25	19.3	1.5
Deerlick 2	382	21.3	408	18.5	2.8

Supplemental Table S5: Spread of data in moon-free nights. Unit: $mag_{SQM}/arcsec^2$

Site name	Clear 15.9%	Clear 84.1%	Clear difference	Overcast 15.9%	Overcast 84.1 %	Overcast difference
IP Feld	21.4	21.2	0.1	21.6	21.4	0.2
Parey	21.4	21.1	0.2	21.2	20.9	0.3
Springendal	21.2	21.0	0.2	21.0	20.1	0.9
Rad Kwk	20.8	20.7	0.1	21.0	19.6	1.4
Arkemheen	20.4	20.1	0.3	20.1	18.4	1.8
CESAR	20.4	19.8	0.5	19.6	18.5	1.1
Potsdam Bb	20.3	20.0	0.3	18.0	17.4	0.6
Arnhem	20.2	19.8	0.4	19.1	17.4	1.7
Nove	19.7	19.2	0.5	19.0	17.2	1.7
Berlin St LU	19.3	19.0	0.3	17.1	16.2	0.9
Schipluiden	18.8	18.1	0.7	17.3	14.9	2.4
Vlaardingen	17.4	17.0	0.4	16.7	15.5	1.2
Deerlick 2	21.5	21.1	0.3	21.9	21.1	0.9

Supplemental Table S6: Spread of data in overcast nights. Unit: $mag_{SQM}/arcsec^2$

Site name	Moonless 15.9%	Moonless 84.1%	Moonlit 15.9%	Moonlit 84.1 %
Schiermnk	21.9	21.3	20.3	17.0
IP Feld	21.6	21.4	20.0	17.8
Parey	21.2	20.9	20.1	17.9
Springendal	21.0	20.1	19.8	17.3
Rad Kwk	21.0	19.6	19.9	17.4
Arkemheen	20.1	18.4	18.5	17.0
CESAR	19.6	18.5	18.5	17.2
Potsdam Bb	18.0	17.4	18.0	17.2
Arnhem	19.1	17.4	17.8	17.0
Vestby	18.2	17.0	18.8	16.7
Nove	19.0	17.2	17.4	16.7
Bremen H-L	17.1	17.0	16.3	16.1
Morehead	17.7	16.1	17.9	15.7
Berlin St LE	16.5	16.4	17.2	16.2
Berlin St LU	17.1	16.2	17.0	16.2
Utrecht	17.8	16.2	17.0	16.0
Schipluiden	17.3	14.9	16.2	15.0
Plymouth Dv	17.5	14.6	16.5	14.6
Madrid UCM	17.9	17.0	16.2	14.8
Vlaardingen	16.7	15.5	16.3	15.5
Plymouth Rm	19.5	18.8	19.1	17.1
Plymouth Mk	19.5	16.7	19.1	17.5
Deerlick 1	21.2	20.7	21.0	17.4
Deerlick 2	21.9	21.1	20.5	17.5

Supplemental Figure S7: Contour plots for midnight cloudy sky radiance at different moon elevations, page 1 of 4

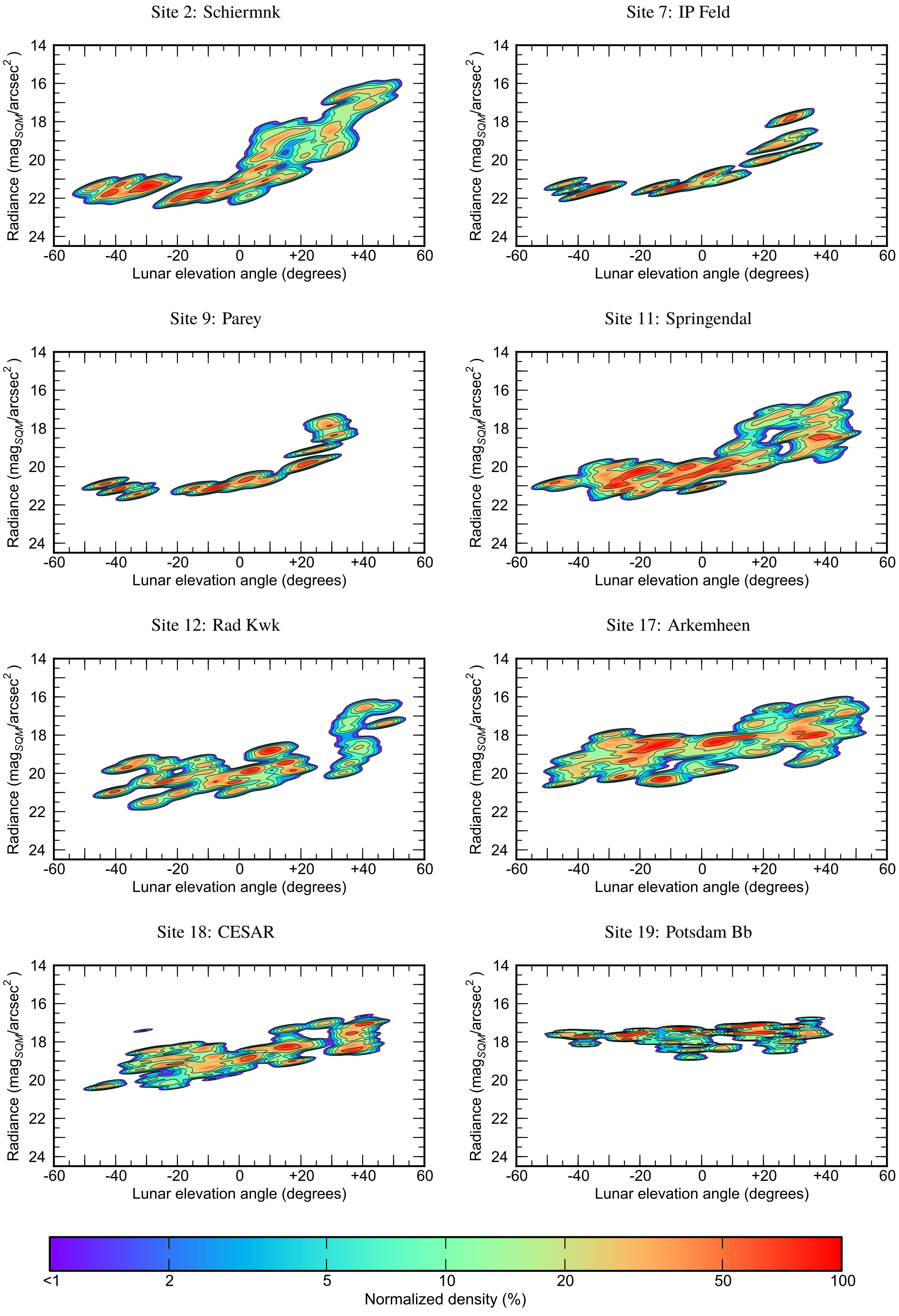

Supplemental Figure S7: Contour plots for midnight cloudy sky radiance at different Moon elevations, page 2 of 4

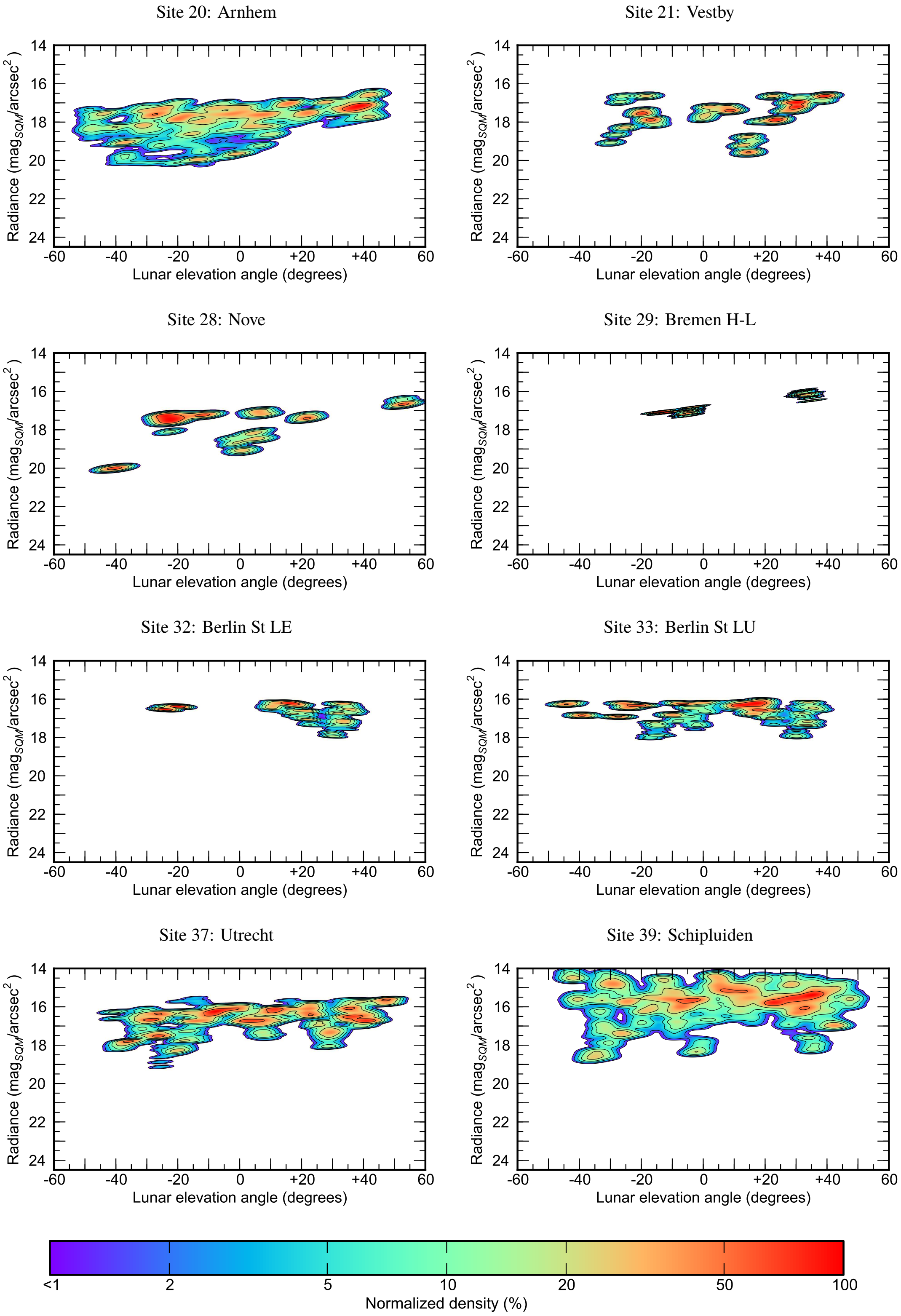

Supplemental Figure S7: Contour plots for midnight cloudy sky radiance at different moon elevations, page 3 of 4

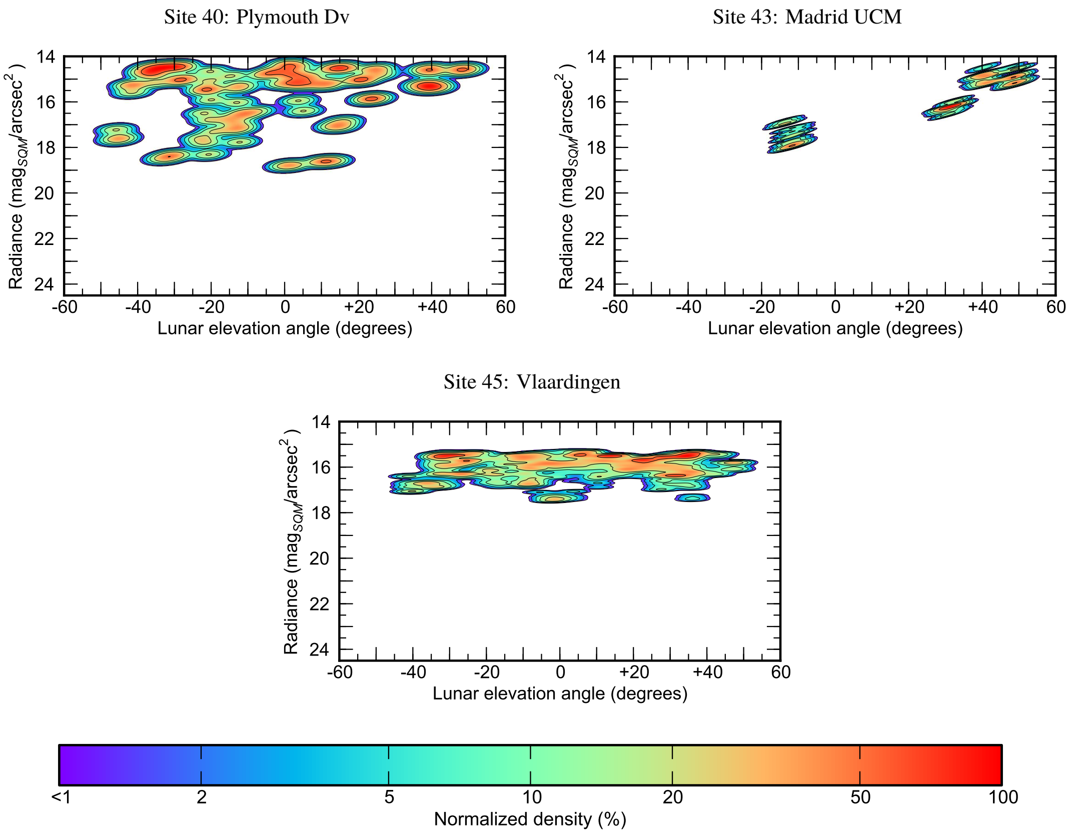

Supplemental Figure S7: Contour plots for midnight cloudy sky radiance at different moon elevations, page 4 of 4

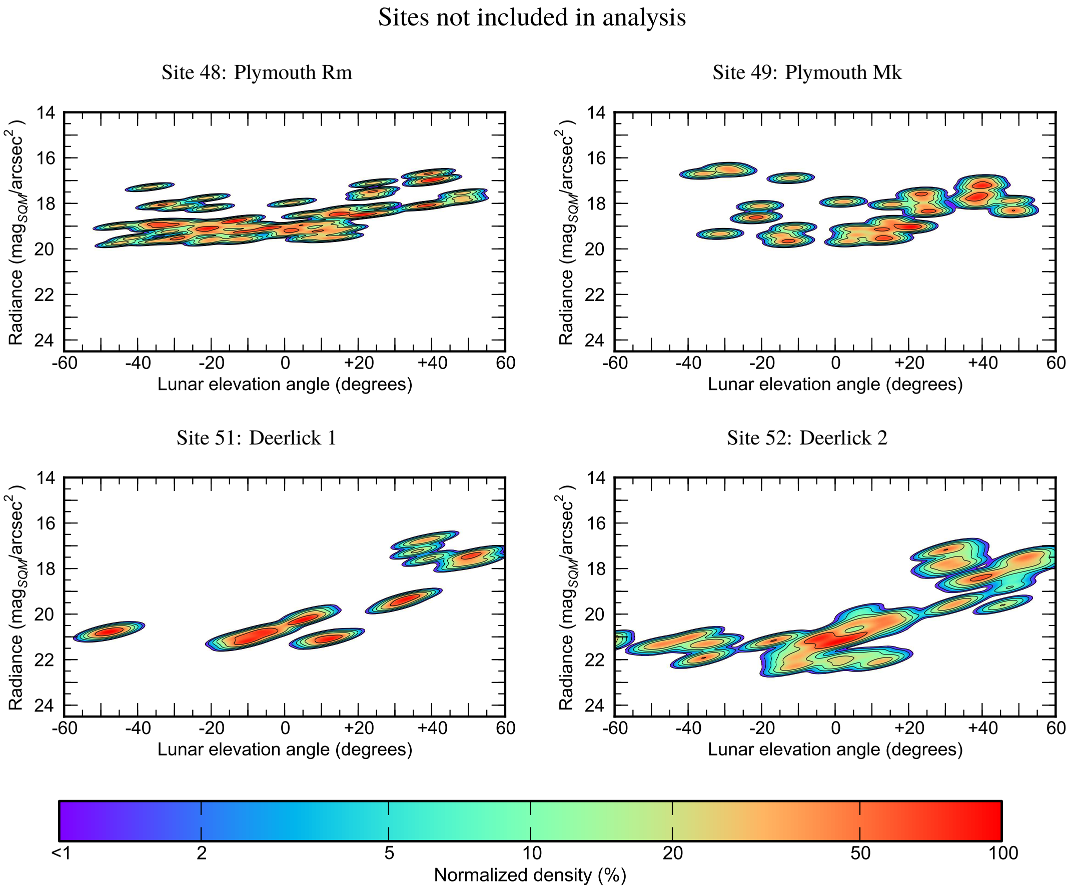

Supplemental Figure S8: Evolution of night sky radiance over the course of the night for each site, page 1 of 7

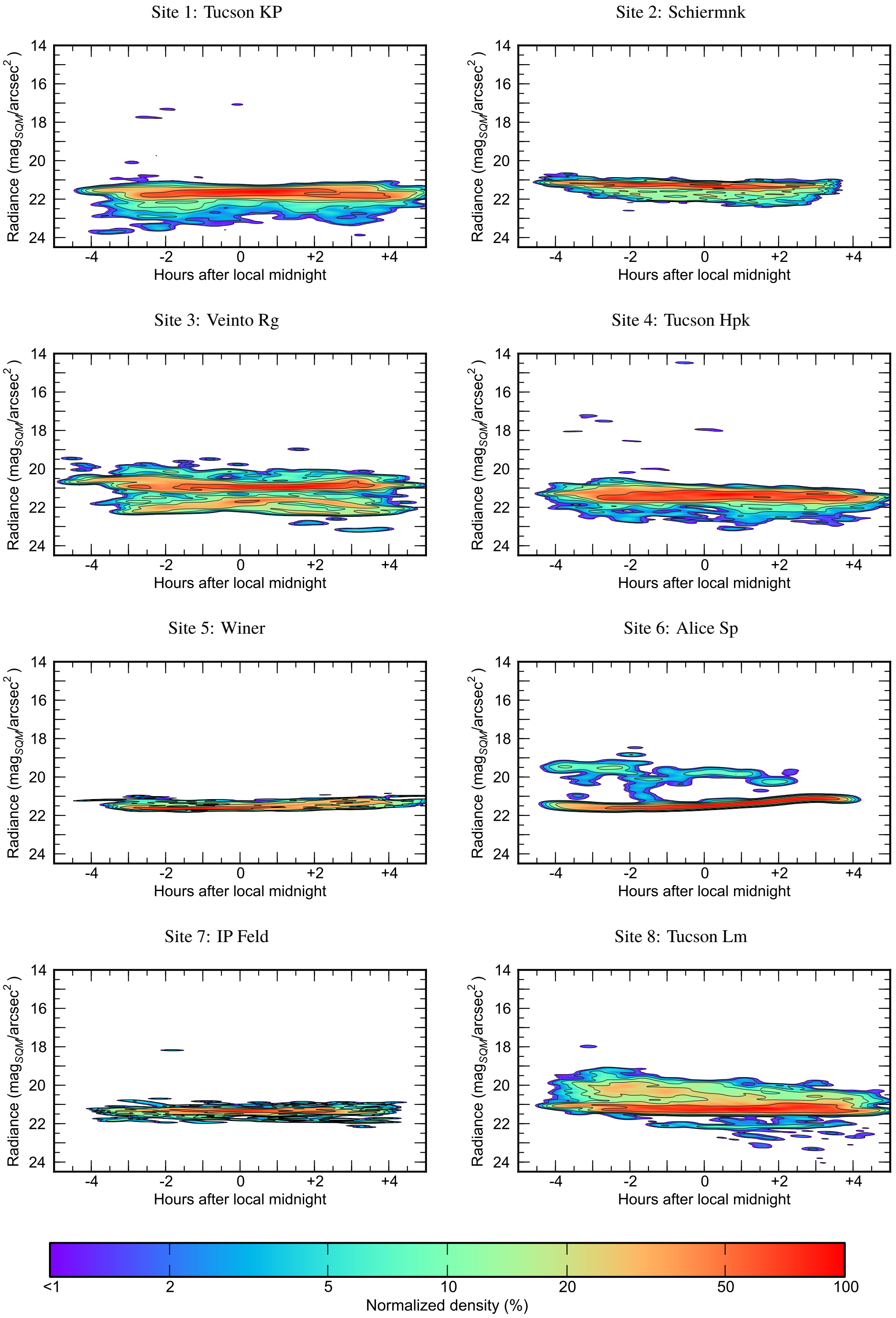

Supplemental Figure S8: Evolution of night sky radiance over the course of the night for each site, page 2 of 7

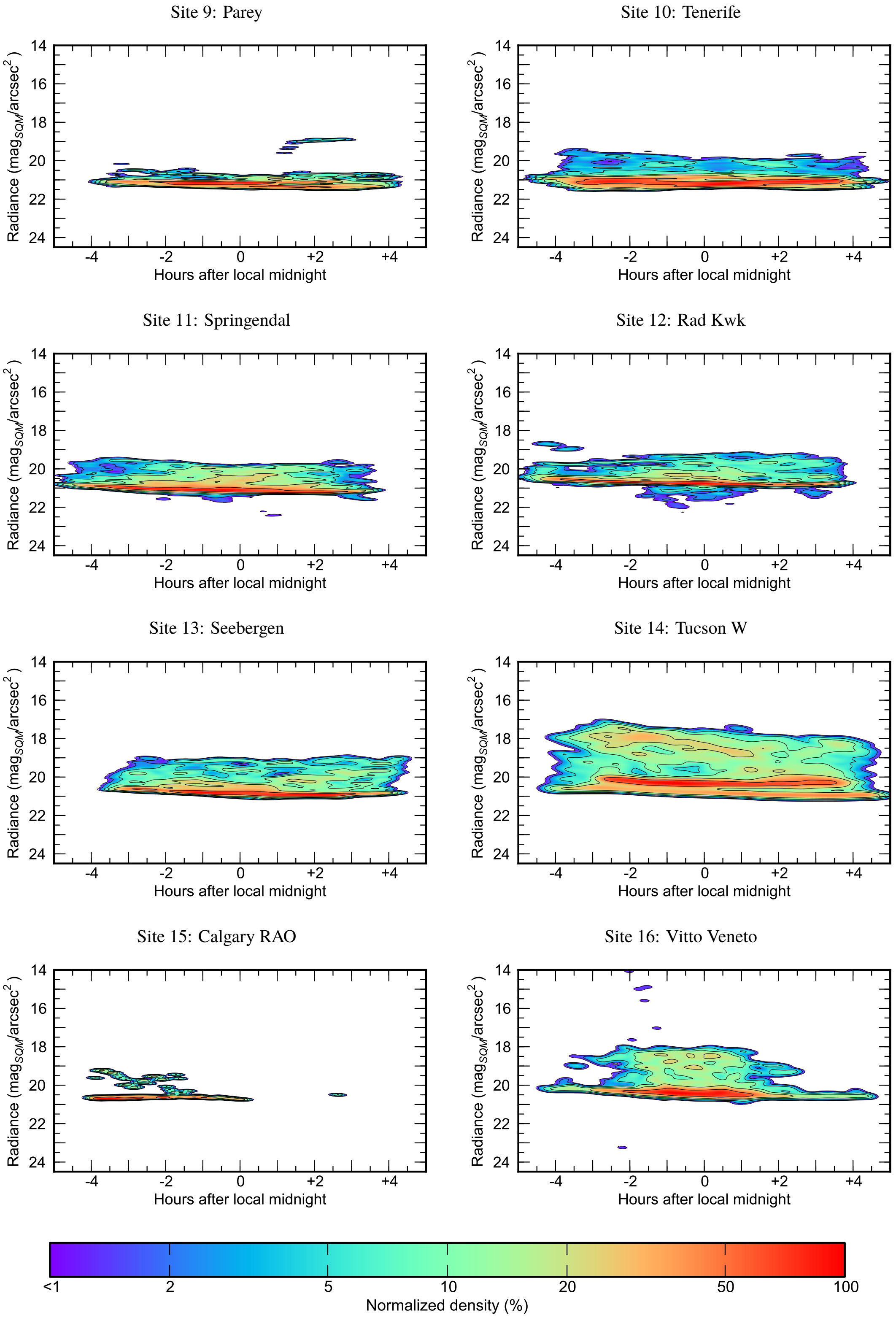

Supplemental Figure S8: Evolution of night sky radiance over the course of the night for each site, page 3 of 7

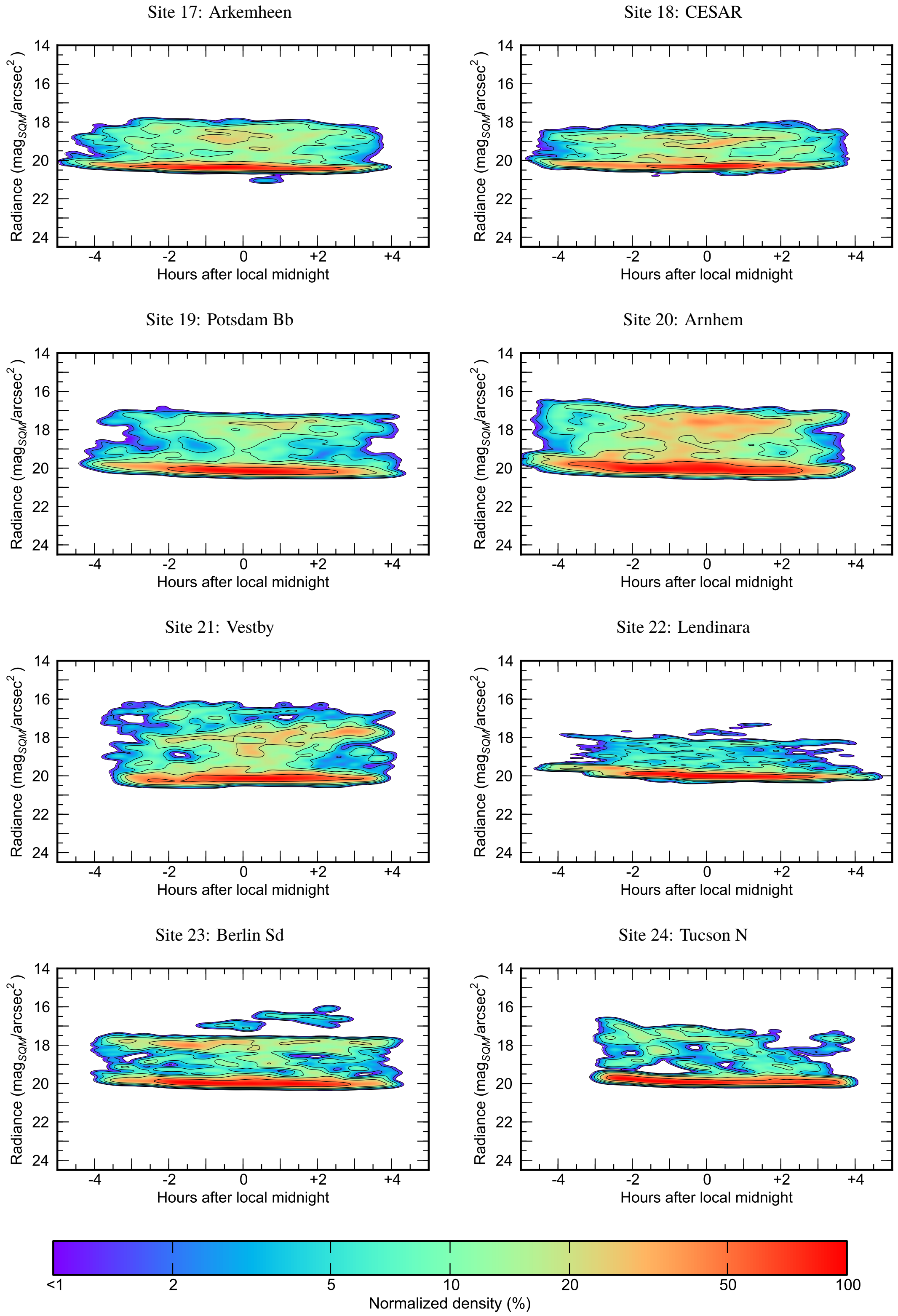

Supplemental Figure S8: Evolution of night sky radiance over the course of the night for each site, page 4 of 7

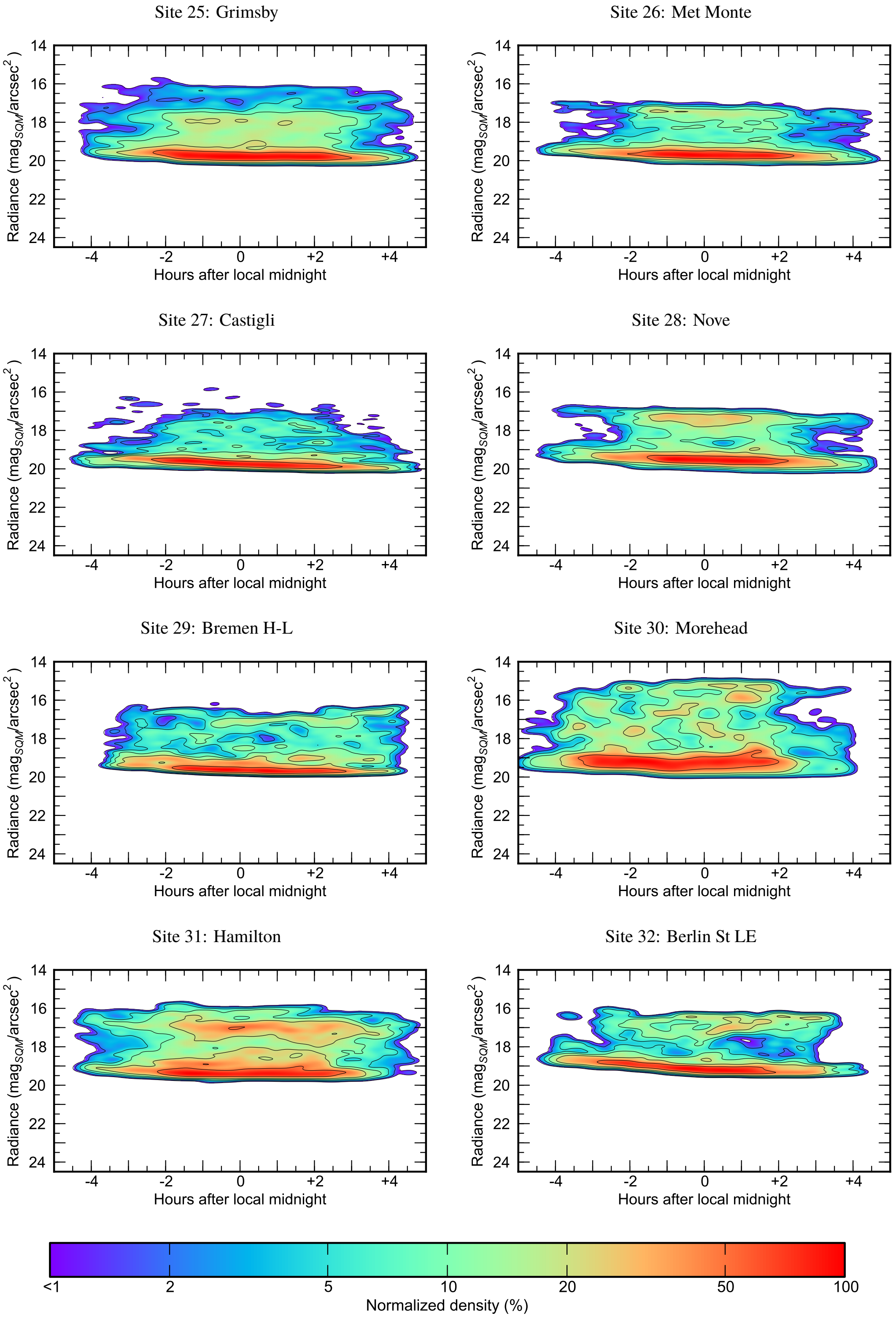

Supplemental Figure S8: Evolution of night sky radiance over the course of the night for each site, page 5 of 7

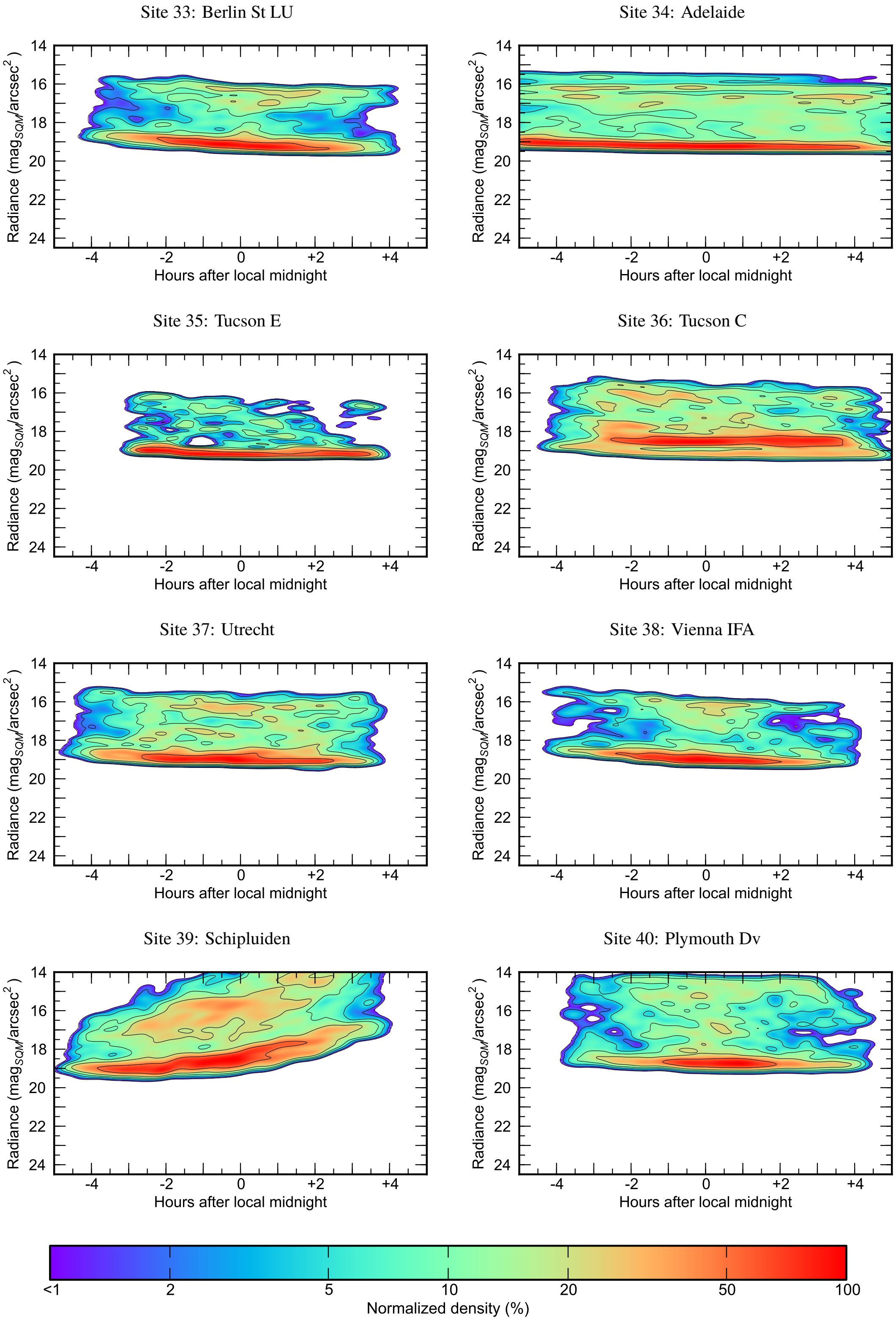

Supplemental Figure S8: Evolution of night sky radiance over the course of the night for each site, page 6 of 7

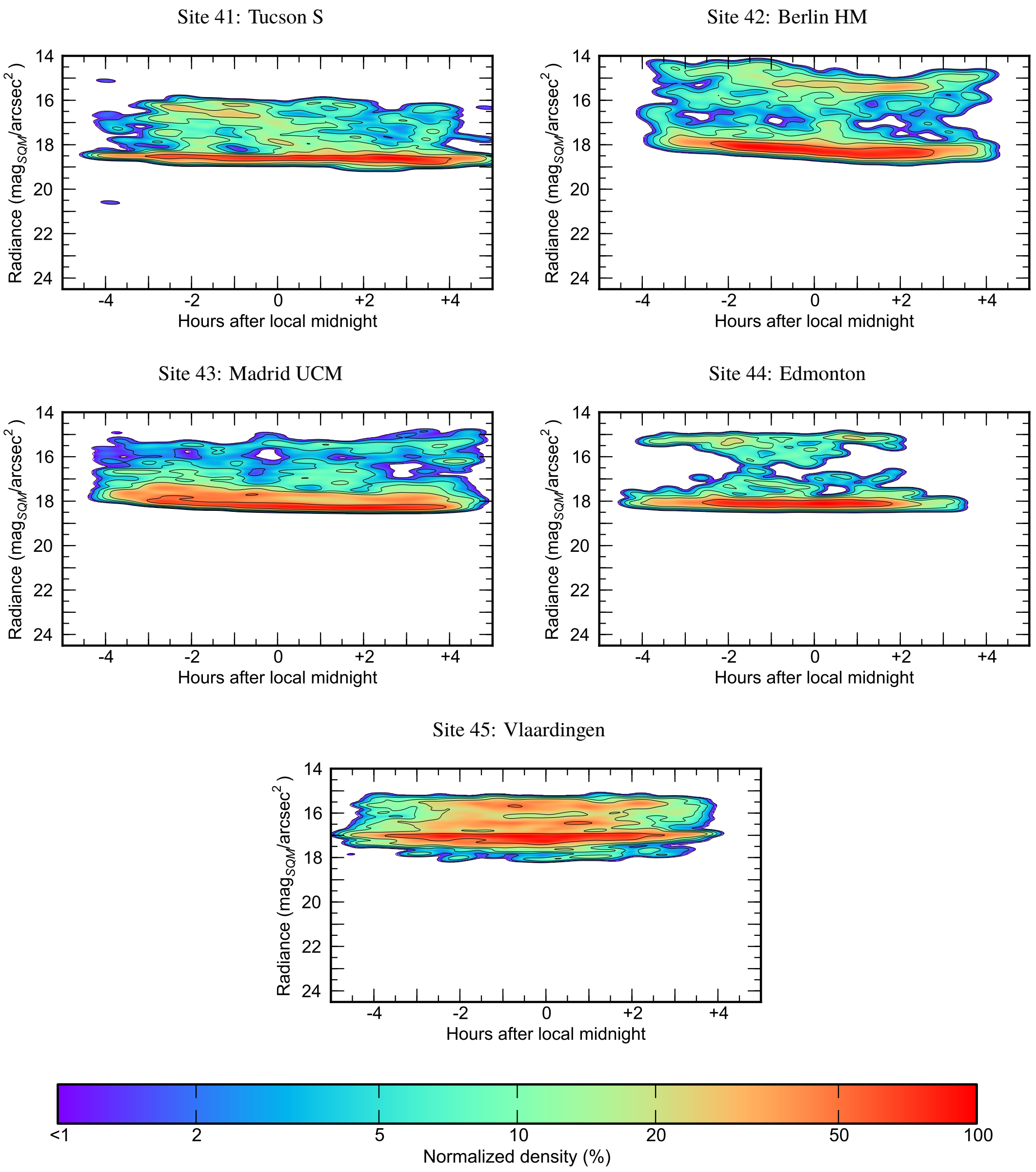

Supplemental Figure S8: Evolution of night sky radiance over the course of the night for each site, page 7 of 7

Sites not included in analysis

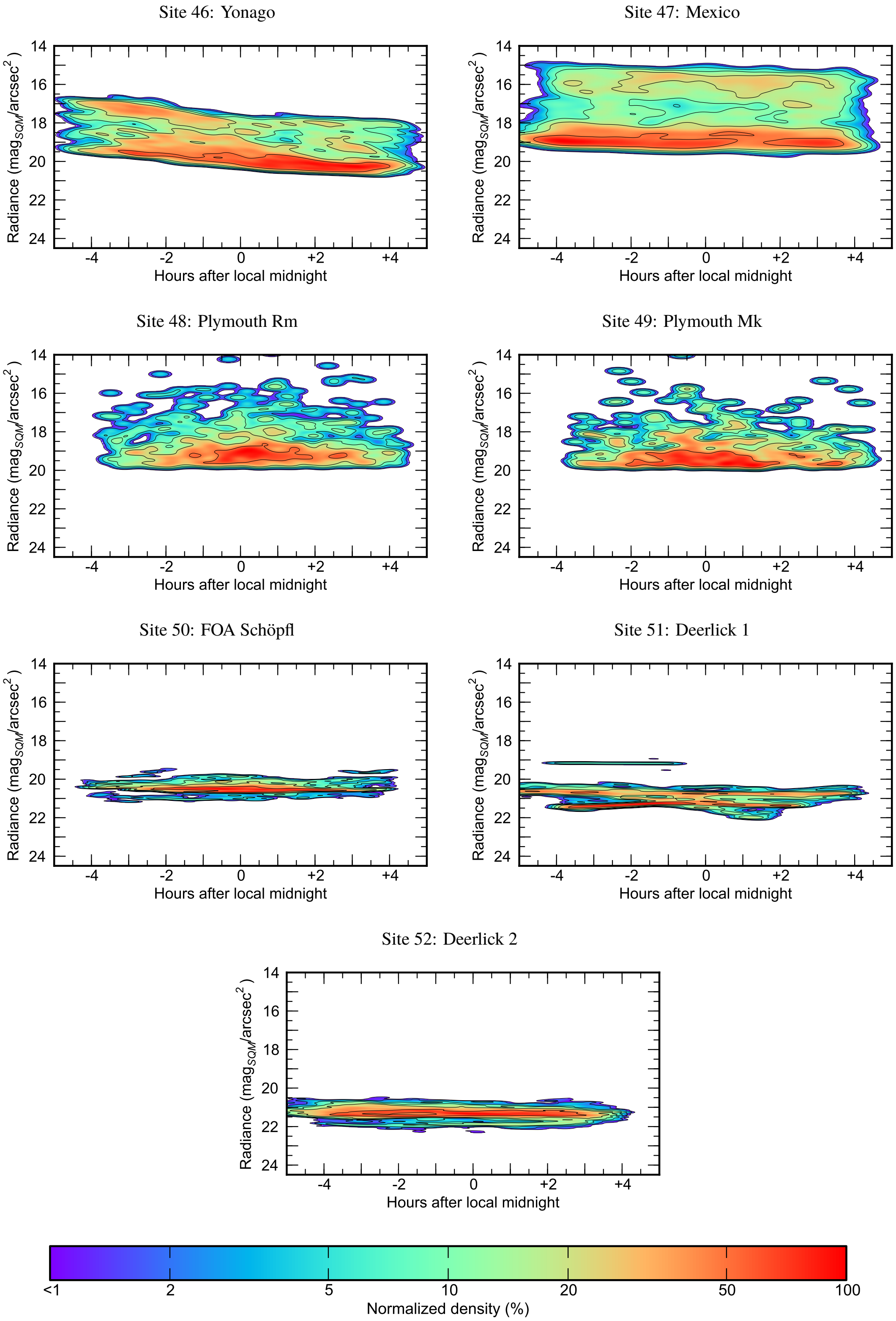

Supplemental Figure S9: Comparison of the change in the artificial sky radiance from 22:00 to midnight on a linear scale

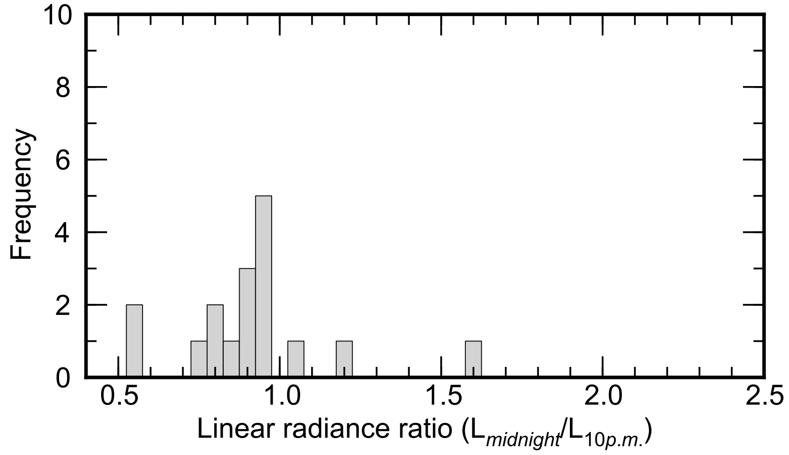

Supplemental Figure S10: Comparison of the change in the artificial sky radiance from midnight to 02:00 on a linear scale

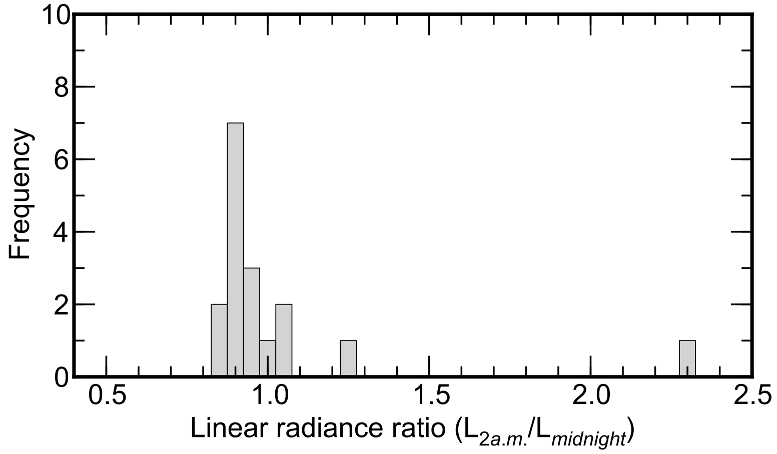

Supplement: Supplementary Information [file srep08409-s1.pdf]
